# Negative affect influences the computations underlying food choice in bulimia nervosa

**DOI:** 10.7554/eLife.105146

**Published:** 2026-10-01

**Authors:** Blair RK Shevlin, Loren Gianini, Joanna Steinglass, Karin Foerde, E Caitlin Lloyd, Kelsey Hagan, Laura A Berner

**Affiliations:** 1 Department of Psychiatry, Icahn School of Medicine at Mount Sinai New York United States; 2 Department of Psychiatry, Columbia University Medical Center New York United States; 3 Department of Psychology, University of Amsterdam Amsterdam Netherlands; 4 Department of Psychiatry, Virginia Commonwealth University Richmond United States; University of Toronto Canada; University College London United Kingdom

**Keywords:** bulimia nervosa, computational psychiatry, decision-making, eating disorders, cognitive modeling, Human

## Abstract

Individuals often consume tasty, calorically dense foods in response to negative emotions, a phenomenon exemplified by notions of ‘stress eating’ and ‘comfort food’. While this link between food and mood can become pathological in binge eating, the decision-making processes underlying this link are poorly understood. Here, we investigated the impact of acute increases in negative affect on when and how strongly the perceived tastiness and healthiness of foods influence food choices in healthy adults and individuals with bulimia nervosa (BN), an eating disorder characterized by cycles of over- and under-consumption of food. In a randomized crossover design, 25 women with BN and 21 healthy controls completed two sessions where they received either a neutral or negative affect induction and then completed a food choice task. Using a time-varying diffusion decision model, we assessed how negative affect influences food choice dynamics for high- and low-fat foods. In the neutral affect condition, individuals with BN considered tastiness relative to healthiness of high-fat foods sooner than healthy controls but maintained a restrictive food choice policy by reducing the weight on tastiness. After a negative affect induction, both groups showed a stronger bias toward considering tastiness before healthiness, but this bias was exaggerated in individuals with BN. This affect-induced bias for high-fat foods predicted more frequent subjective binge episodes over 3 months. These results provide insights into how negative emotion influences food choices and may explain why binge eating in BN is more likely during high negative affect, while dietary restriction is more likely during low negative affect.

## Introduction

Every day, multiple times a day, people must make decisions about what to eat. A growing body of research suggests that these decisions involve considerations of different attributes of the available food options (e.g., healthiness and tastiness), and that these attributes often present the decision-maker with challenging conflicts (e.g. , ‘Should I choose the healthier, less delicious food or the better tasting, less healthy food?’). Difficulties resolving these conflicts can take on pathological forms in individuals with eating disorders, and some eating disorders are characterized by food choices that are biased toward one extreme (e.g., toward subjectively less tasty, but healthier, low-fat foods in anorexia nervosa; Foerde et al., 2015). However, food choices seem to episodically oscillate *between* extremes in the case of bulimia nervosa (BN), which is commonly marked by prolonged periods of rigid dietary restriction punctuated by out-of-control binge eating of highly palatable foods. Although subclinical forms of this oscillation are ubiquitous in examples of ‘yo-yo dieting’ and ‘dietary lapses’, surprisingly little cognitive neuroscience research to date has attempted to explain it.

Self-report data suggest that rising negative affect is one reliable predictor of the switch from more to less restrictive food choices (Konttinen et al., 2010). Although some individuals reduce intake in response to stress (Hill et al., 2022), research using both animal models and healthy adult samples show that stress can precipitate the overconsumption of highly palatable foods (Dionysopoulou et al., 2021; Jacques et al., 2019; Tomiyama, 2019). This connection between negative emotion and food choice is even more pronounced in binge eating. Inducing negative affect in the laboratory environment disproportionately increases food intake among individuals with binge eating (Telch and Agras, 1996). Ecological momentary assessment data show that binge eating in the natural environment is also much more likely to occur in the context of increasing and unstable negative emotions (Alpers and Tuschen-Caffier, 2001; Berg et al., 2013; Haedt-Matt and Keel, 2011; Hilbert and Tuschen-Caffier, 2007; Smyth et al., 2007). In addition, reactivity to negative emotions, or negative urgency, has been shown to predict or correlate with more frequent binge eating in individuals with BN (Fischer et al., 2013; Schnepper et al., 2021).

Although momentary increases in negative emotions are closely tied to the overconsumption of highly palatable foods, the cognitive processes underlying this tight link, particularly in the case of binge eating, are not well understood. Studies to date have failed to find support for the notion that increased negative affect, specifically stress, impairs inhibitory control processes in healthy adults (Allen et al., 2022) or in BN (Dreyfuss et al., 2017; Westwater et al., 2021). While another study found that healthy adults and individuals with BN differed in how stress impacted visual processing-related neural activity, they failed to find support for the hypothesis that increased negative affect abnormally increased limbic neural responses to palatable food stimuli in BN (Collins et al., 2017).

**Figure 1.**
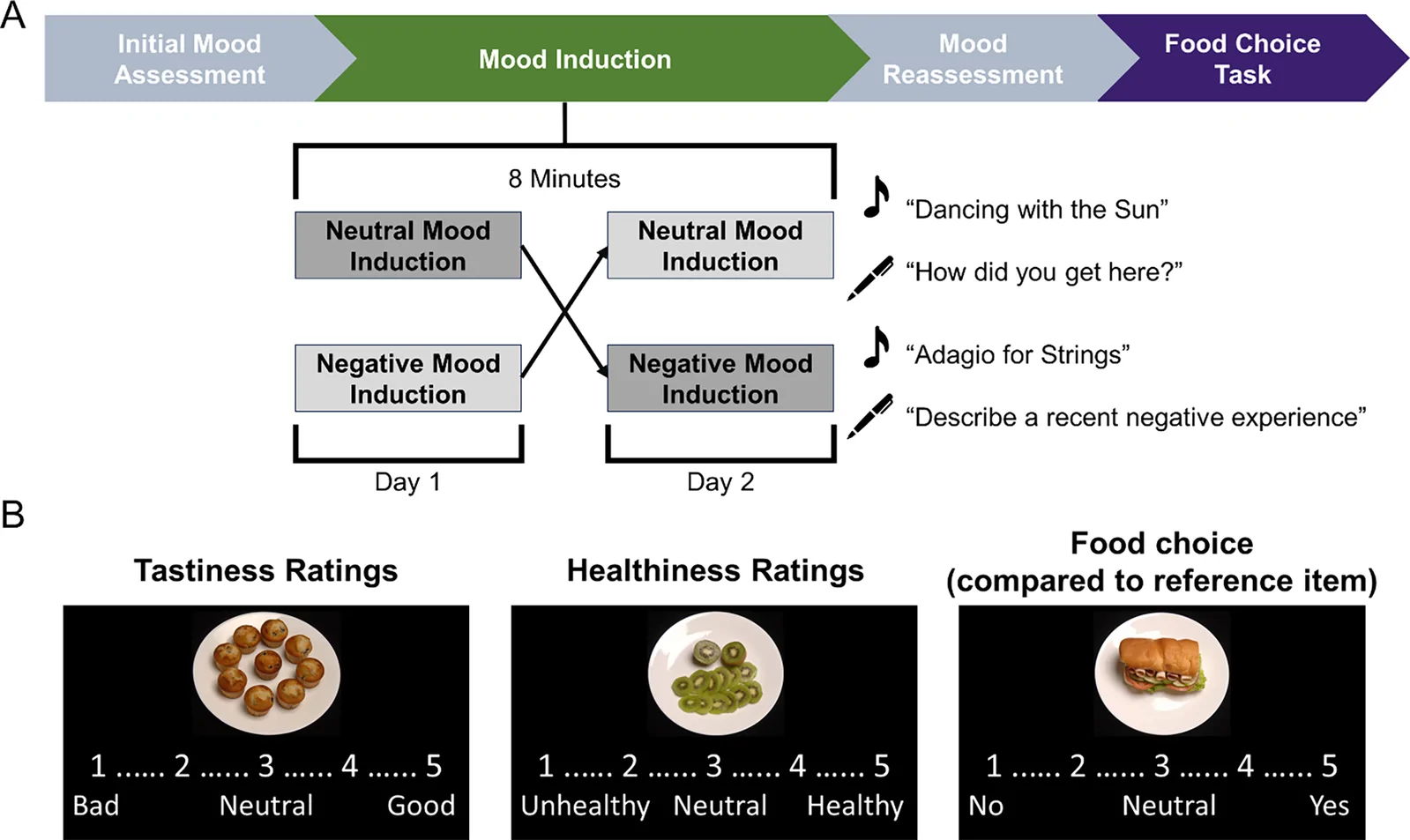
Affect induction and task design. (**A**) **Study timeline**. BN and HC participants completed the study tasks on two separate days. On session 1, participants were randomly assigned to the neutral mood or negative mood induction before completing the Food Choice Task. On session 2, participants experienced the alternative mood induction before completing another run of the Food Choice Task. The mood inductions involved combinations of music and autobiographical writing. (**B**) **Food Choice Task**. During the Food Choice Task, both BN and HC participants rated 43 food items across three phases. In the Tastiness Ratings and Healthiness phases, participants rated each item on a 5-point Likert scale from Bad to Good and Unhealthy to Healthy, respectively. In the Choice phase, participants indicated their strength of preference for a presented food item, compared to a personally tailored neutral reference item.

**Figure 2.**
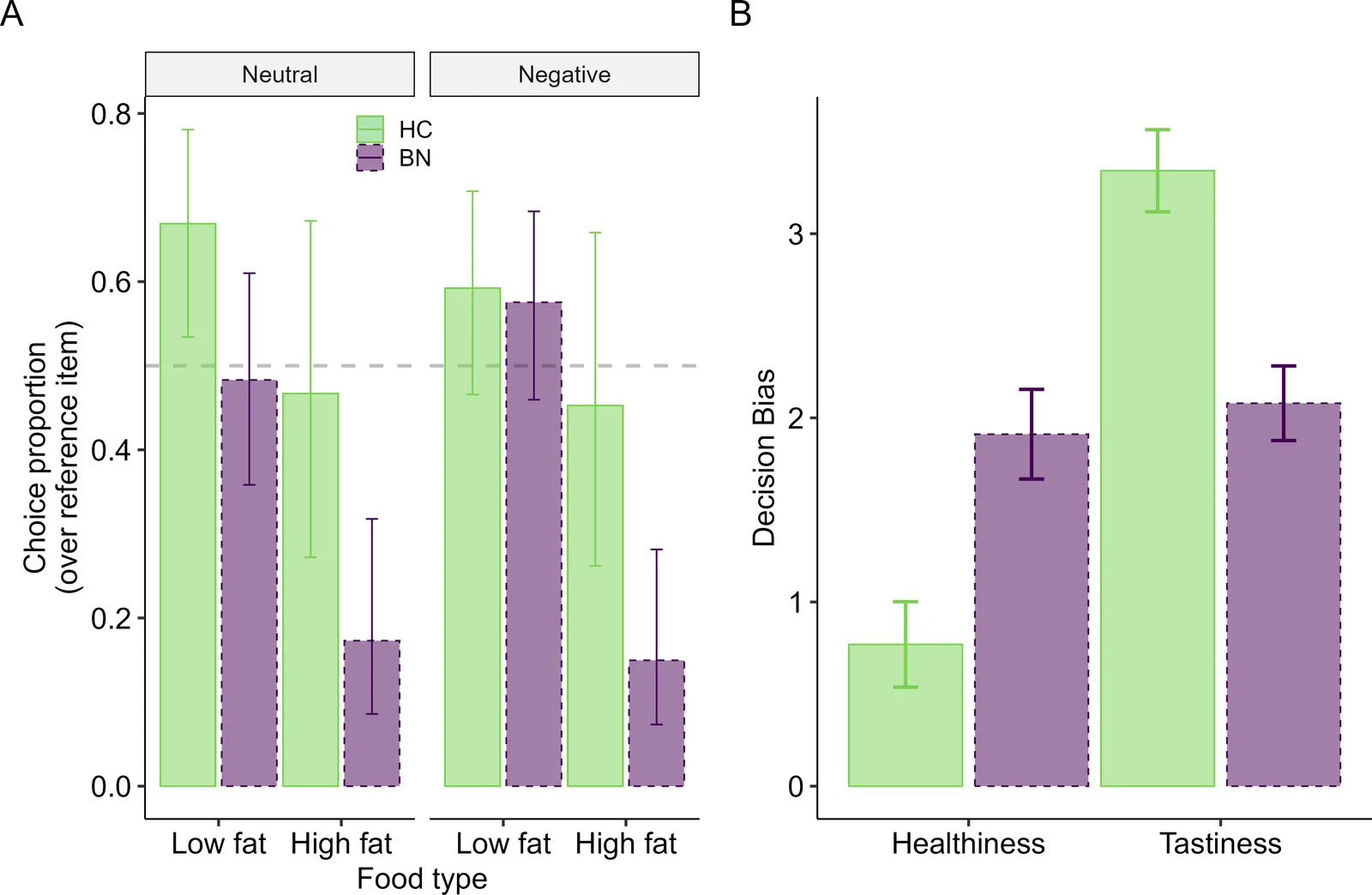
Food Choice Task behavior. (**A**) **Choice behavior estimated from regression models**. Re-analysis of the raw data excluding outlier response times (2.5% of trials *n* = 85) replicated original findings (Gianini et al., 2019). While both groups were less likely to choose high-fat foods (over the neutral reference item) than low-fat foods (over the neutral reference item), the BN group was even less likely than the HC group to choose high-fat food items. However, we did not identify any significant effects of the Affect Condition on choices. Error bars indicate 95% confidence intervals of the estimated effects. (**B**) **Influence of health and taste ratings on food choice**. Health ratings influenced food choice more in the BN group than in the HC group. Within the HC group, food choice was influenced more strongly by taste ratings than health ratings. Error bars indicate standard errors of the estimated coefficients. *Note*. Corresponding statistics are presented in Appendix 1—table 9. HC = healthy controls (*n* = 21); BN = bulimia nervosa (*n* = 25).

We recently tested the possibility that negative affect abnormally impacts decision-making in BN by promoting food choices that include the high-fat, appetitive foods that are typically consumed during binge-eating episodes (Gianini et al., 2019). Individuals with BN and healthy controls completed a food choice task after a negative and neutral affect induction (Figure 1). Counter to expectations, negative affect did not influence ultimate food choices in either group, and women with BN showed restrictive food choices whether neutral or negative affect was induced: they chose low-fat foods more often (Figure 2A), and these choices were strongly predicted by healthiness ratings (Figure 2B). However, these analyses focused primarily on individuals’ ultimate choices and may have overlooked the possibility that even small changes in negative affect could influence more subtle aspects of the food decision-making process leading up to those choices.

**Figure 1—figure supplement 1.**
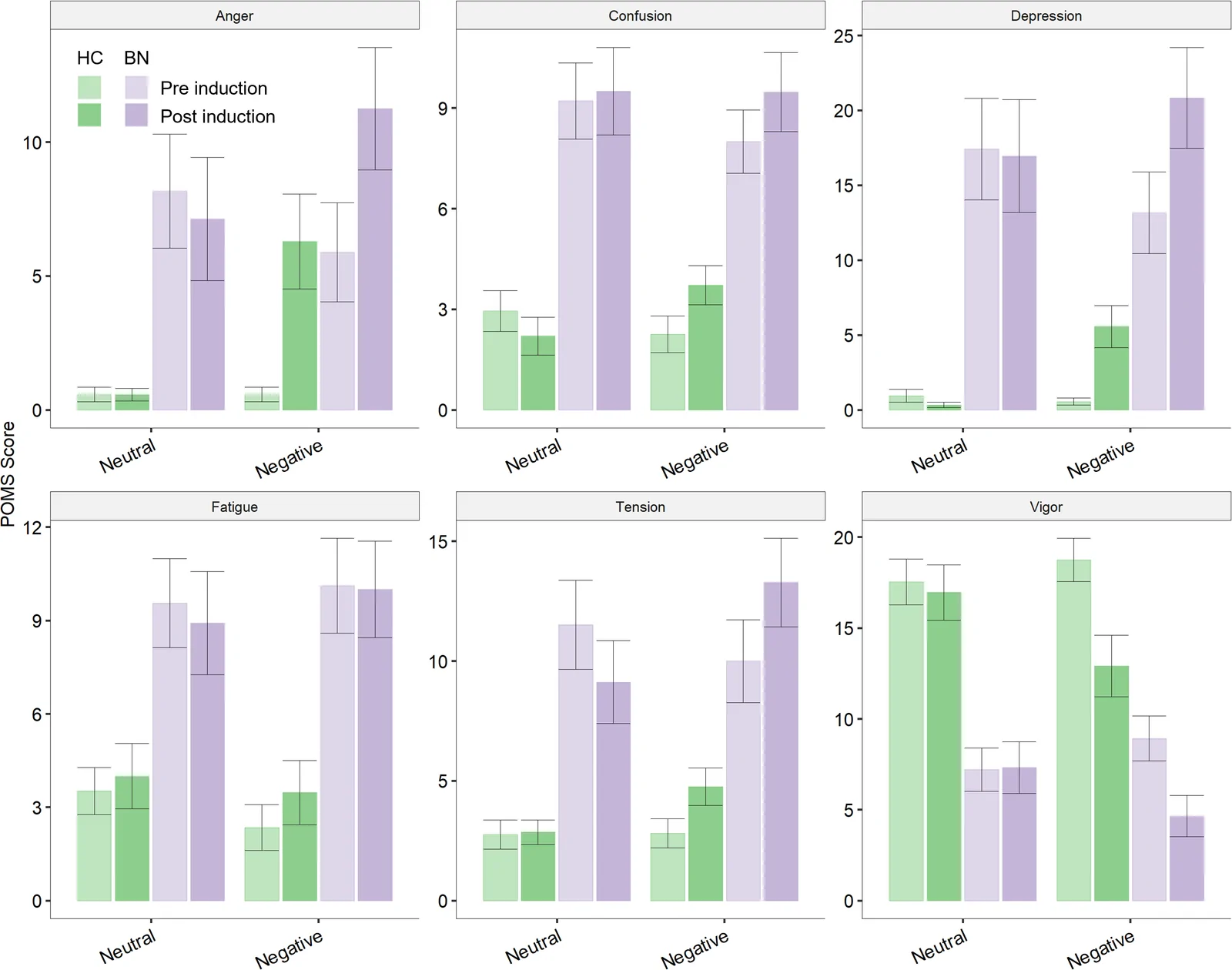
Effectiveness of the affect induction across POMS subscales. Change in each Profile of Mood States (POMS) subscale (Anger, Confusion, Depression, Fatigue, Tension, Vigor) following the neutral and negative affect inductions, shown separately for the healthy control (HC; *n* = 21) and bulimia nervosa (BN; *n* = 25) groups. Error bars indicate standard errors of the mean.

These more subtle features can be quantified with computational cognitive modeling. Sequential sampling models leverage both choice and response time data to estimate parameters that capture individual differences in decision-making dynamics (Forstmann et al., 2016; Ratcliff and Smith, 2004). These models assume that decision-making involves the noisy accumulation of evidence over time until a predetermined threshold is reached, leading to a response. Therefore, both ultimate responses and response times are meaningful signals about the cognitive processes underlying decision-making. Though originally developed to describe perceptual, psychomotor, and memory tasks, these models can be applied to value-based decision-making tasks by incorporating participants’ ratings of different attributes for the stimuli presented in a trial. This allows researchers to examine how and when these attributes influence the decision-making process (Busemeyer et al., 2019; Clithero, 2018).

Research using sequential sampling models indicates that food choices are influenced not only by the weights assigned to attributes like healthiness and tastiness, but also by the time at which these attributes enter the decision-making process (Chen et al., 2022; HajiHosseini and Hutcherson, 2021; Hutcherson and Tusche, 2022; Maier et al., 2020; Sullivan et al., 2015; Sullivan and Huettel, 2021). Specifically, using time-varying sequential sampling models, studies have demonstrated that healthy individuals assign different subjective weights to healthiness and taste attribute ratings of foods (i.e., they have differing degrees to which they influence the evidence accumulation rate); that healthiness and taste attribute information can enter the decision process at different times; and that these relative weighting strengths and timing of attribute consideration have distinct influences on ultimate food choices (Maier et al., 2020). This notion is consistent with theories of both emotion- and food-craving regulation, which posit that there are separate attention deployment and stimulus valuation steps involved in these regulation processes (e.g., Giuliani and Berkman, 2015; Gross, 2015; Han et al., 2018).

**Figure 3.**
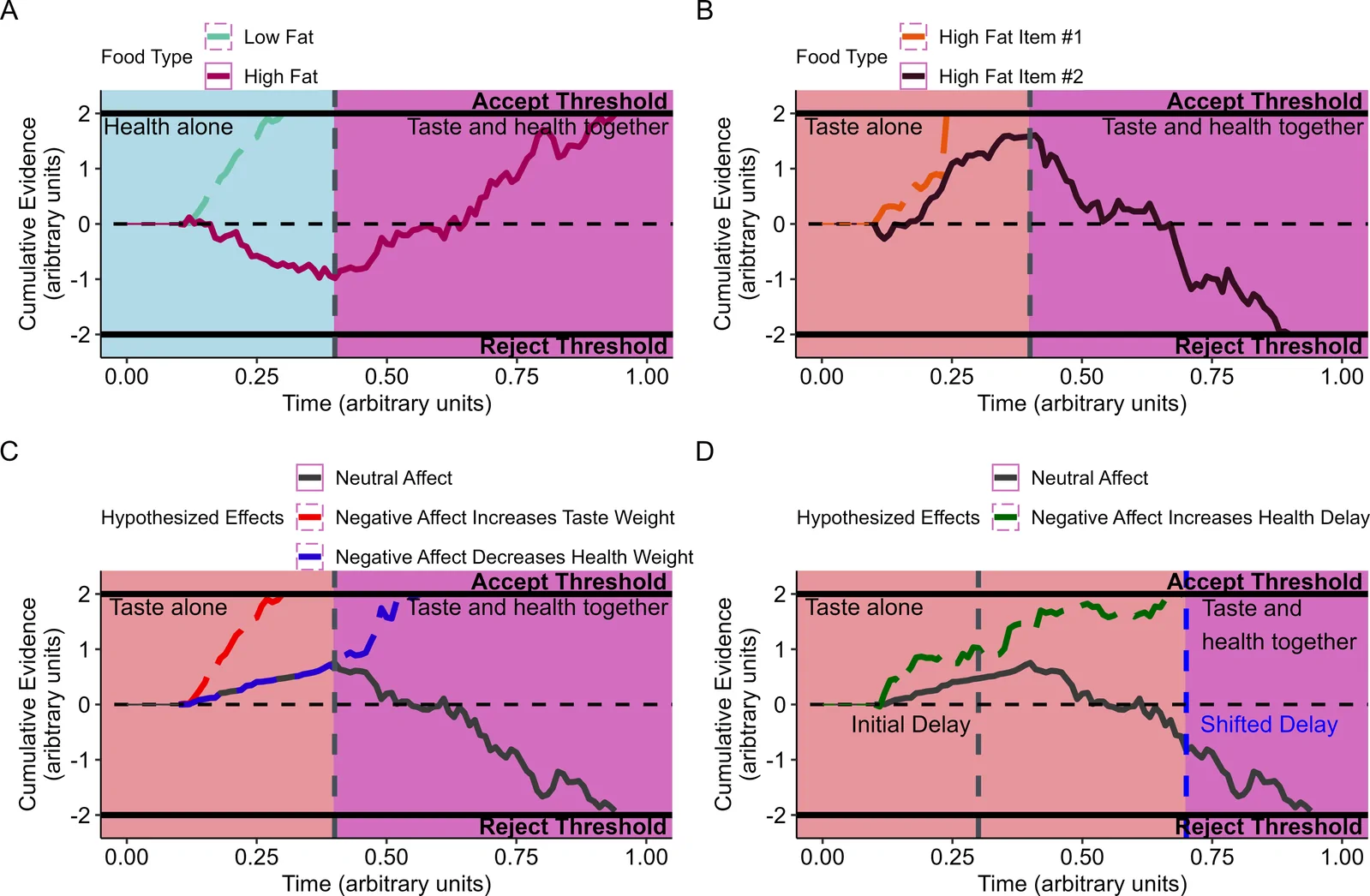
Example evidence accumulation trajectories predicted by the starting time diffusion decision model (stDDM). Each trajectory is a simulated agent that considers one attribute alone before beginning to consider both tastiness and healthiness attributes together. (**A**) **Tastiness onset delay**. The dashed, green trajectory represents a case where positive healthiness information about a low-fat food item quickly biases the agent toward the accept threshold before taste information has time to influence the evidence accumulation process. The solid purple trajectory illustrates a case where evidence related to a high-fat food is initially biased toward the reject threshold while the aversive healthiness information dominates the evidence accumulation process. Once information about the item’s appetitive tastiness comes online, the evidence accumulation changes its trajectory toward the accept threshold. (**B**) **Healthiness onset delay**. The dashed, orange trajectory illustrates a case where evidence related to the highly appetitive tastiness attribute dominates the evidence accumulation process, influencing the trajectory to terminate at the accept threshold of a high-fat food before the healthiness attribute is considered. For the solid brown trajectory, a less appetitive high-fat food item is ultimately rejected once aversive healthiness information enters the evidence accumulation process. (**C, D**) **Hypothesized model of affect-induced binge-eating**. Each trajectory is a simulated agent with BN making a decision involving a high-fat food. During neutral affect (gray solid line), the high-fat food is trending toward the accept threshold until the onset of aversive healthiness information biases the trajectory toward the reject threshold. (**C**) **Attribute-weight hypothesis**. During negative affect, either the attribute weight for taste information increases (red dashed line) or the attribute weight for healthiness information decreases (blue dashed line). In both cases, the trajectory is ultimately biased toward the accept threshold. (**D**) **Attribute-onset hypothesis**. During negative affect, the initial delay shifts and taste information is accumulated longer before healthiness information comes online. With a longer delay in the onset of healthiness information, the evidence accumulation for the high-fat food has enough time to reach the accept threshold.

Notably, even if one of these decision parameters (e.g., weights on healthiness or tastiness, attribute onsets) biased individuals toward selecting a low-fat (or high-fat) food, the other parameter could compensate for it to ultimately generate the opposite response (Figure 3). For example, if taste information was considered before healthiness information, but healthiness information was weighted strongly enough, a decision-maker could choose a healthier, instead of a tastier, food (Figure 3B). These latent aspects of the decision-making process, either orthogonally or in parallel, could be sensitive to state changes like increases in negative affect and could be altered in BN. Specifically, these latent decision parameters could help explain why dysregulated consumption of tasty, high-fat foods in BN is more likely during periods of high negative affect, but restricted intake of lower-fat foods is more likely during periods of low negative affect. In one case, increasing negative affect could alter an individual’s attribute weights, either reducing their weight on healthiness (dashed blue line) or increasing their weight on tastiness (dashed red line), which would increase their likelihood of choosing binge-type foods that are considered to be highly palatable yet also unhealthy (Figure 3C). Alternatively, increasing negative affect could cause individuals with BN to consider healthiness information later in the decision-making process, giving taste information more time to bias choices toward binge-type foods (Figure 3D). Clarifying these mechanisms is critical for translational efforts because data suggest that the weighting of attributes and the time at which they are considered have different neural substrates (Maier et al., 2020), and the treatment offered to patients should depend on which scenario more accurately reflects their pathology.

Here, we applied a diffusion decision model (DDM) with a time-varying drift rate to food choice and response time data from our controlled, randomized crossover study in women with BN and healthy controls (Figure 1A). This model allowed us to investigate the potential exaggerated influence of negative affect on the dynamics of food-specific decision-making in BN. Specifically, we tested how food type (low-fat/high-fat) and affective state (neutral/negative) influenced decision-makers’ attribute weights and onsets.

Our hypotheses were derived from the clinical phenomenology underlying BN. As outlined above, individuals with BN seem to vacillate between unstable decision dynamics: many individuals with the disorder maintain a relatively restrictive diet until negative emotional states induce binge-eating episodes and subsequent compensatory behaviors (i.e., purging). Given this instability, we predicted that our BN participants’ food choices would be influenced by components of the decision-making process whose opposing dynamics could be disrupted by increasing negative affect.

We first hypothesized that in states of neutral affect and for high-fat foods, one of these opposing dynamics would compensate for the other to promote ultimate choices that are restrictive. This could be achieved by either (1) weighing taste information more strongly but considering it later than healthy controls, or (2) weighing tastiness less strongly but considering it earlier than healthy controls. Second, we hypothesized that, in BN, increasing negative affect would abnormally impact at least one of these latent aspects of decision-making to increase the bias toward high-fat foods. Even though prior analyses indicate that high-fat foods were not ultimately chosen more often, we predicted that after a negative affect induction, individuals would either (1) be slower to consider healthiness information, but still weight taste information just as strongly, or (2) they would put less weight on healthiness information, but still consider taste information sooner.

Because these combinations require one feature of the decision process to compensate for another, they would result in a much less stable dynamic compared to one in which information weights and onset times are concordant. For example, when healthiness information is considered sooner *and* is more strongly weighted than taste information, foods low in healthiness would almost always be rejected. In contrast, when healthiness information is considered later but is more strongly weighed than taste information, food choice behavior can be more easily perturbed with state-based changes in weights or the delay in attribute onsets. Here, we show evidence for these unstable decision dynamics that can account for a bias in BN toward low-fat, healthier foods in states of relatively low negative affect, but toward high-fat, tastier foods in states of high negative affect. Although negative affect delayed the onset of the consideration of healthiness information among both HC and BN participants, this effect was more pronounced in the BN group. Ultimately, we found that although individuals with BN consistently weighed healthiness information more strongly, negative affect delayed its entry into the evidence accumulation process. With this longer delay, taste information has more time to bias decision-makers toward high-fat food choices before healthiness information can come online.

## Results

### Negative affect aberrantly delays consideration of healthiness information among women with BN

We analyzed data from individuals with BN (*n* = 25) and healthy controls (HC, *n* = 21) who completed a computerized Food Choice Task following negative and neutral affect inductions, across two visits (Figure 1). Mood was assessed before and after the affect inductions using the Profile of Mood States (POMS; McNair et al., 1971). Negative affect scores were calculated by summing the five negative subscale scores (e.g., anger, confusion, depression, fatigue, and tension) and subtracting the positive subscale score (e.g., vigor). Negative affect scores increased significantly for both groups following the negative affect induction (Gianini et al., 2019, reproduced in Appendix 1—table 1). Critically, post-induction negative affect within the BN group was significantly higher following the negative affect induction than after the neutral affect induction (mean difference = 17.40, SE = 4.21, *t* = 4.13, p < 0.001, Cohen’s *d* = 0.83; see Appendix 1 for full details), confirming that BN participants completed the food decision task under meaningfully distinct affective states across the two sessions.

To quantify the specific computations performed during Food Choice Task decision-making, we used a time-varying DDM (Ratcliff and McKoon, 2008). This time-varying model introduces a starting time parameter that indicates the delay with which different attributes enter the evidence accumulation process (Chen et al., 2022; Lombardi and Hare, 2021; Maier et al., 2020). Using this starting time DDM (stDDM), we can determine how individuals weigh healthiness versus taste information and when those attributes influence food-related decision-making in BN and HC groups in each affective state. The stDDM was fitted to data that included individual-level response times, choices, z-scored healthiness and tastiness ratings, and affect induction.

We specified a stDDM where individual parameters were drawn from four separate hyperparameters that varied both by group (BN or HC) and by condition (neutral or negative affect induction). To test our hypotheses about the interactions between affect and food type (low-fat or high-fat) on food choice, we allowed each attribute weight parameter $\left (\omega _{Taste}\,\mathrm{and} \,\omega _{Health}\right)$ and the relative-starting time parameter $\left (\tau _{s}\right)$ to vary as a function of food type. The drift rate determining the evidence update can be written as follows:

(1) $$\displaystyle \nu(t)= \begin{cases} \omega_{Taste}\cdot VD_{Taste} & \text{If } \tau_s\lt0 \land 0\ltt\lt|\tau_s| \\[4pt] \omega_{Health}\cdot VD_{Health} & \text{If } \tau_s>0 \land 0\ltt\lt\tau_s \\[4pt] \omega_{Taste}\cdot VD_{Taste} +\omega_{Health}\cdot VD_{Health} & \text{If } t\gt|\tau_s| \end{cases}$$

where $\omega _{Taste}$ is the subjective weight given to tastiness, $\omega _{Health}$ is the subjective weight given to healthiness, $VD_{Taste}$ and $VD_{Health}$ are the value differences in taste and healthiness attribute ratings, respectively, and $\tau _{s}$ is the time at which the taste and healthiness attributes come into the evidence accumulation process. If $\tau _{s}$ > 0, healthiness information is accumulated first, and evidence from taste comes into consideration at time $\tau _{s}$, whereas $\tau _{s}$ < 0 means that taste information is accumulated first and evidence from healthiness information starts to come into the decision process at time $|\tau _{s}| $. To test our hypotheses, medians of the posterior distributions for subject-level parameters were used as the dependent variables in our statistical analyses. We ran three separate, fully factorial (Group-by-Affect Condition-by Food Type), mixed-effects linear regressions, one for each parameter of interest. Each of these binary categorical variables was contrast coded using the values –1 and 1, allowing us to separately assess group differences across condition.

Results exploring additional parameters (boundary separation $\left (\alpha \right)$, non-decision time $\left (\tau _{ND}\right)$, and starting point bias $\left (z\right)$) are presented in the Appendix 1 along with model comparisons, model identifiability, posterior predictive checks, and parameter recovery exercises.

**Figure 4.**
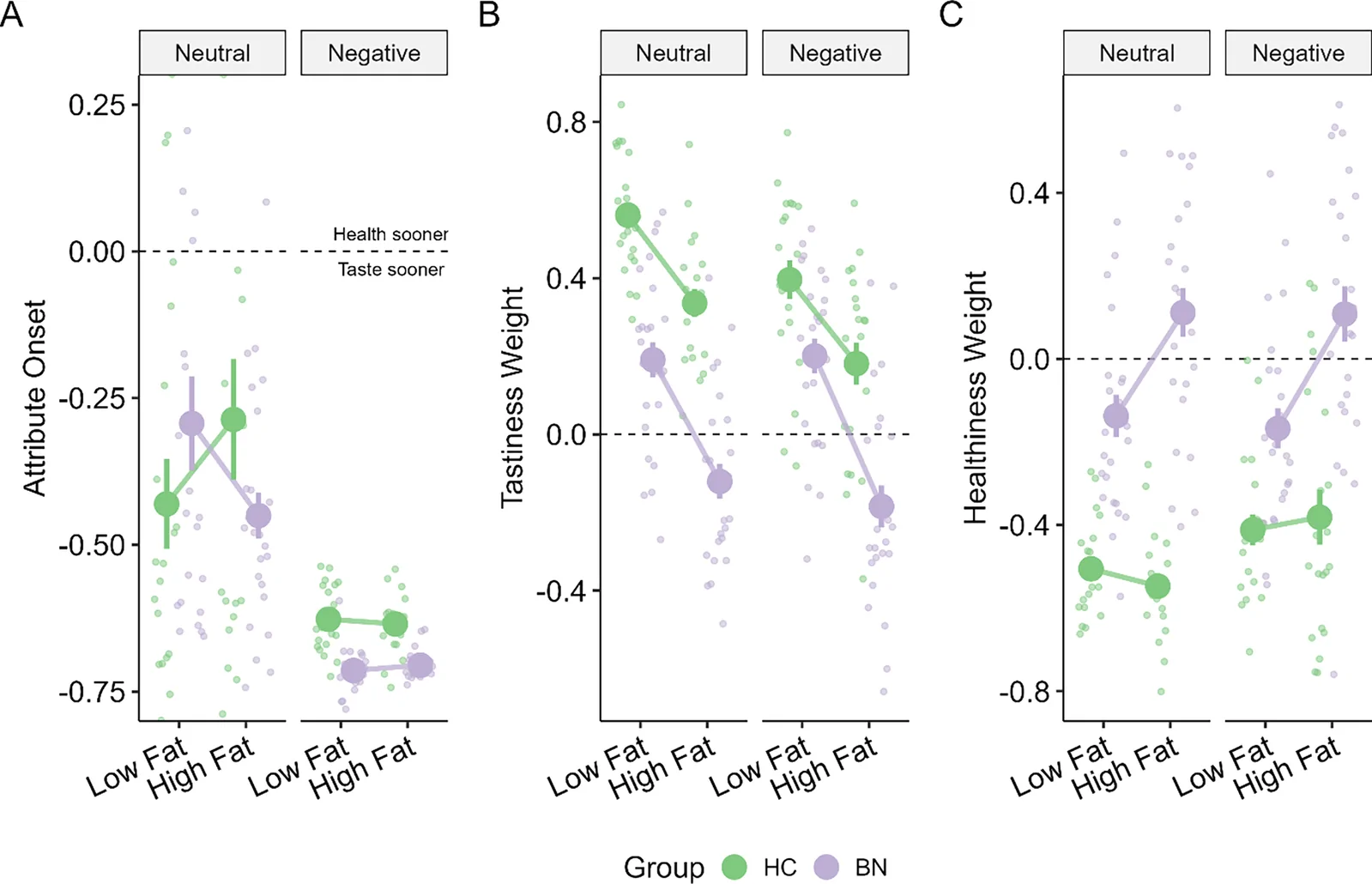
Parameter estimates. Colors indicate diagnosis: purple = bulimia nervosa (BN; *n* = 25); green = healthy controls (HC; *n* = 21). Error bars represent the standard error of the mean. (**A**) **Attribute onset**. In the neutral condition, we observed a Group by Food Type cross-over effect: while the BN group showed a greater initial bias toward accumulating tastiness information of high-fat foods than low-fat foods, the HC group showed a greater tastiness information bias for low-fat foods than high-fat foods. After the negative affect induction, any Food Type-based distinctions disappeared, and both groups’ biases toward tastiness information increased, but this effect was more pronounced in the BN group. (**B**) **Weight on tastiness information**. The HC group put more weight on tastiness information than the BN group. The negative affect induction reduced tastiness weights for the HC group, but not the BN group. (**C**) **Weight on healthiness information**. The BN group put more weight on healthiness information than the HC group, especially for high-fat foods. The negative affect induction did not have significant effects on healthiness weights for either group.

First, we assessed how relative start time parameters varied across groups as a function of food type in each condition. We found a significant three-way interaction between group, condition, and food type (Appendix 1—table 2; $\beta =0.32,t=2.23,\rm p=0.028$), indicating that the two groups differed in how negative affect influenced their attribute bias patterns across food types. To confirm the presence of a three-way interaction, we calculated a difference-in-difference score for each participant’s $\tau _{s}$ parameter: (negative condition, high-fat − negative condition, low-fat) − (neutral condition, high-fat − neutral condition, low-fat). A Wilcoxon rank-sum test comparing these scores between groups confirmed that BN participants showed significantly larger food-type-specific changes in attribute onset timing following negative affect induction relative to HC (*W* = 156, p = 0.018). Visual inspection of the data suggested this interaction was driven by group differences across food types in the neutral condition that disappeared in the negative affect condition, and by a more pronounced influence of negative affect on attribute biases in BN (Figure 4A). Specifically, in the neutral condition, the HC group showed greater taste bias for low-fat versus high-fat foods (*M* = –0.456 to –0.314; Appendix 1—table 3), while individuals with BN showed greater taste bias for high-fat versus low-fat foods (*M* = –0.314 to –0.462; pairwise difference *t*_132_ = –2.33, p = 0.022). The negative affect induction eliminated this crossover pattern, increasing taste biases across both food types and groups, but with greater increases observed in the BN group (Appendix 1—tables 2 and 3).

**Figure 4—figure supplement 1.**
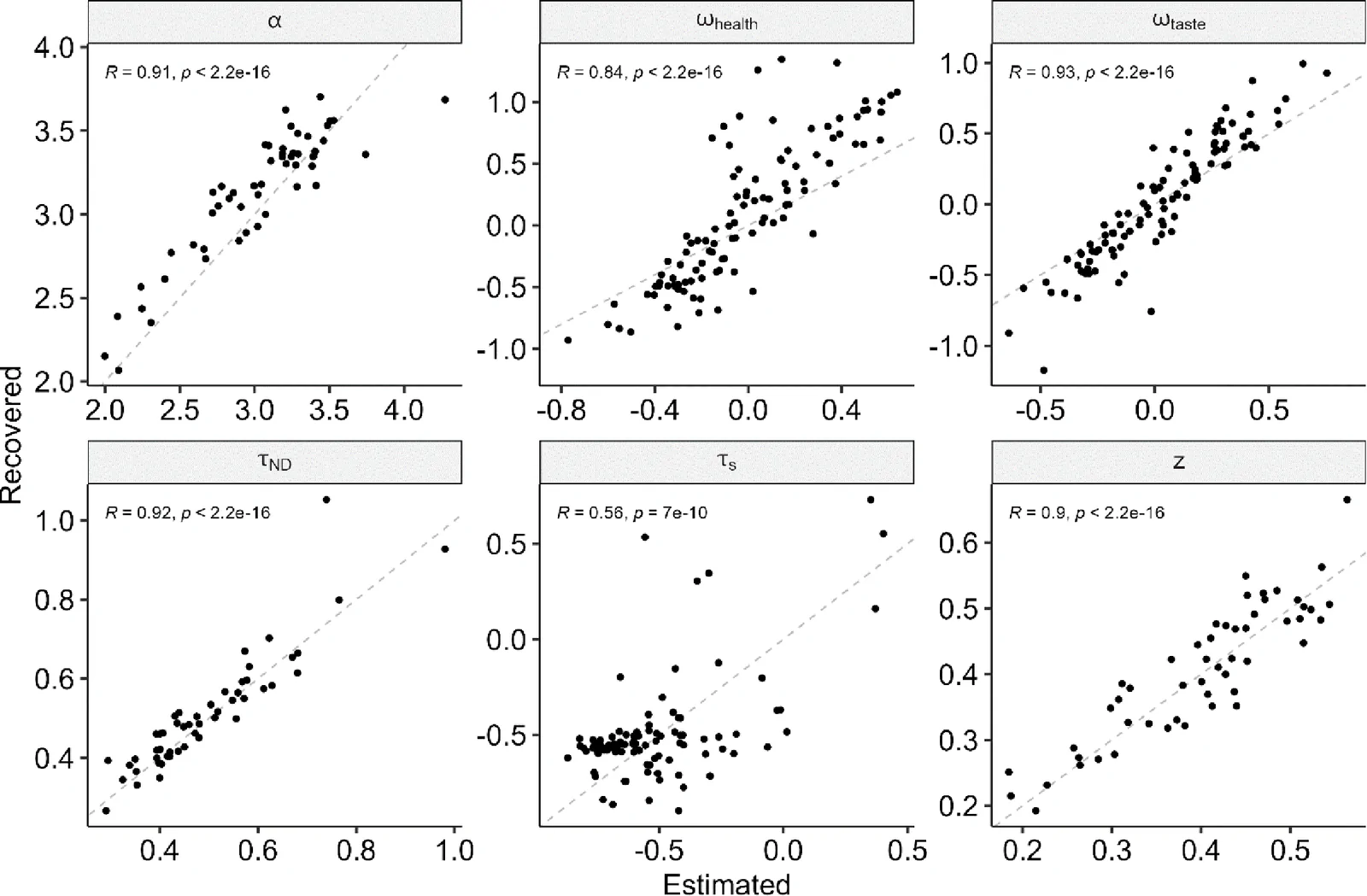
Results from the parameter recovery exercise. Scatterplots relate parameters estimated from the empirical data (fit) to those recovered from data simulated with the winning model (rec), for boundary separation (*α*), healthiness and tastiness attribute weights (*ω*_*health*_, *ω*_*taste*_), non-decision time (*τ*_*ND*_), relative starting time (*τ*_*s*_), and starting point bias (*z*).

### Reduced tastiness weights are abnormally unaffected by negative affect in BN

Our second and third regression models assessed how each attribute weight varied across groups as a function of food type in each condition (Figure 4B, C). For taste weights, the HC group placed consistently higher weight on taste compared to the BN group (Appendix 1—tables 4 and 5; *β* = –0.39, *t* = –6.86, p < 0.001; all pairwise contrasts p ≤ 0.002), and both groups placed less weight on taste for high-fat versus low-fat foods (*β* = –0.23, *t* = –4.05, p < 0.001; all pairwise contrasts p < 0.001). A significant interaction between group and condition (*β* = 0.18, *t* = 2.46, p = 0.015) reflects divergent responses to negative affect: HC showed a significant reduction in taste weighting following the negative affect induction (*t*_132_ = 3.20, p = 0.002), but the BN group did not (*t*_132_ = 0.61, p = 0.542). Consequently, the group difference in taste weighting, while significant in both conditions (all pairwise contrasts p < 0.001), was numerically smaller in the negative affect condition (*M* = 0.21) than in the neutral condition (*M* = 0.39; interaction: *t*_132_ = 1.95, p = 0.053). In contrast, healthiness weights were insensitive to negative affect induction across both groups. However, a significant interaction between group and food type (Appendix 1—tables 6 and 7; *β* = 0.28, *t* = 3.09, p = 0.002) indicated that the BN group placed disproportionately higher weights on health information for high-fat relative to low-fat foods (*t*_132_ = –4.40, p < 0.001), while the HC group showed no difference in health weighting across food types (*t*_132_ = 0.01, p = 0.897).

### More severe symptoms of BN are linked to affect-induced delays in processing healthiness information

**Figure 5.**
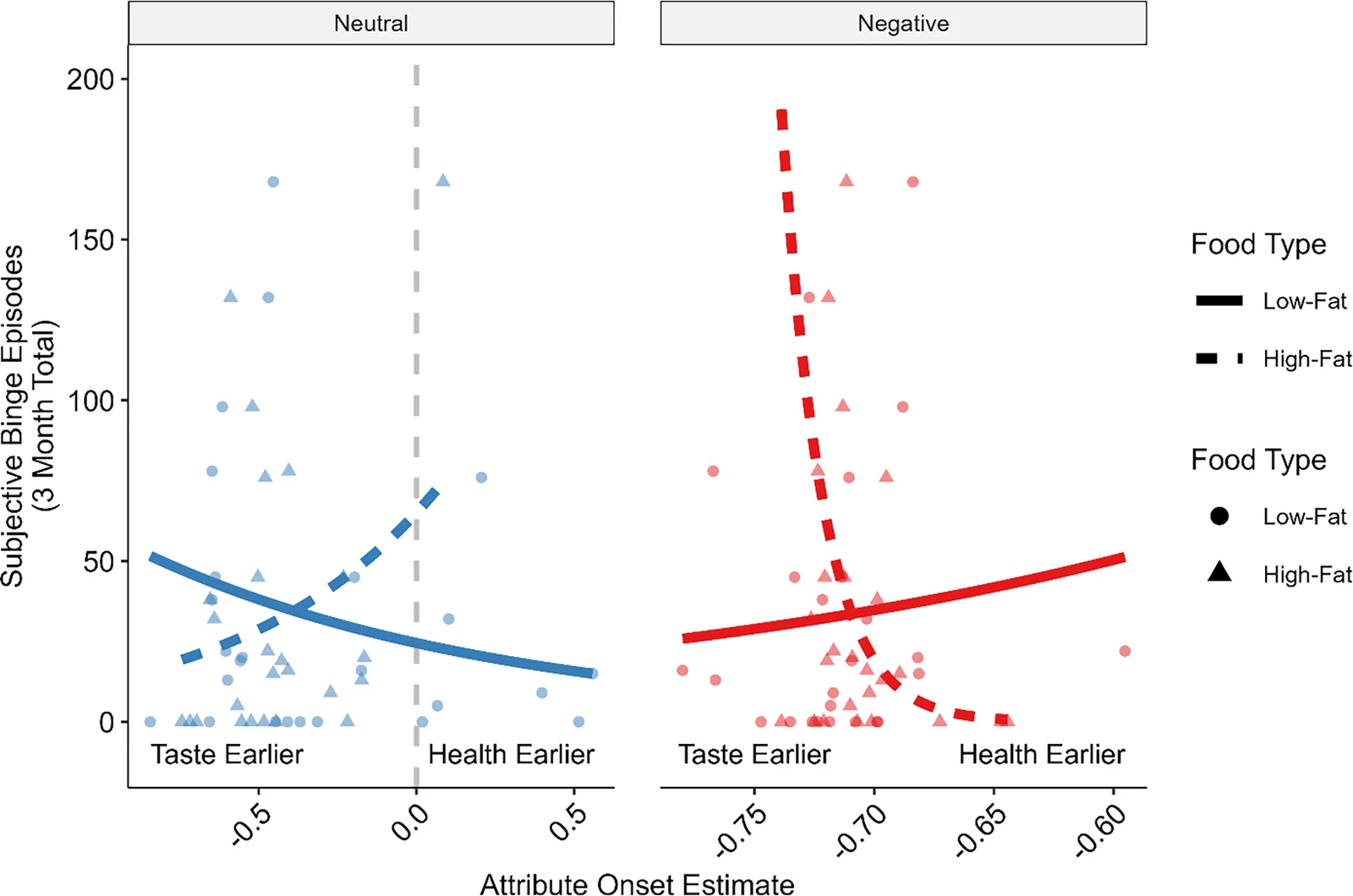
Affect-induced changes in information onset were associated with more frequent subjective binge episodes. In the negative affect condition (right facet), longer delays in accumulating healthiness information (i.e., reduced $\tau _{s}$) for high-fat foods compared to low-fat foods were associated with more frequent subjective binge episodes. Line type and shape refer to Food Type: Solid lines and circles = low-fat foods; dashed lines and triangles = high-fat foods.

To assess the clinical significance of our findings, we sought to connect stDDM parameters to retrospective self-reported symptom severity. For these exploratory analyses, we used median values of the individual-level posterior distributions of the attribute onset parameter $\tau _{s}$, the parameter which showed an exaggerated response to the affect induction in the BN group. These parameter values were then separately added to mixed-effects negative binomial regression models with Group and Food Type to assess their association with two symptom severity measures in the BN group: the frequency of objectively large binge eating episodes (OBEs) and subjectively large binge eating episodes (SBEs) in the past 3 months. We examined both types of out-of-control eating episodes because they both contribute to a diagnosis of BN according to ICD-11 (World Health Organization, 2022), and some data suggest that SBEs are more strongly related to negative affect (Brownstone et al., 2013; Brownstone and Bardone-Cone, 2021; Fitzsimmons-Craft et al., 2014). Indeed, we found that greater negative affect-based decreases in $\tau _{s}$ for high-fat foods relative to low-fat foods were associated with more frequent SBEs in the past 3 months (Appendix 1—table 8; $\beta =- 46.39,t=- 2.80,\rm p=0.005$). In other words, those who focused for longer on the tastiness of high-fat foods after the negative affect induction reported more SBE episodes in the past 3 months (Figure 5). Conversely, affect-based changes in $\tau _{s}$ were unrelated to OBE frequency (Appendix 1—table 8).

## Discussion

Negative affect is a known precursor to overeating and dietary rule-breaking (Frayn and Knäuper, 2018). Healthy adults, and even rodents, can exhibit dysregulated food consumption and altered food choices after periods of stress or other negative emotions (Dionysopoulou et al., 2021; Jacques et al., 2019; Konttinen et al., 2010; Tomiyama, 2019). Negative affect’s influence on eating behavior is particularly pronounced among individuals with clinical diagnoses like BN, who tend to have large and out-of-control eating episodes in states of strong negative emotion (Cardi et al., 2015; Wonderlich et al., 2022). Prior studies have demonstrated that outside of binge-eating episodes, and in states of lower negative affect, individuals with BN typically engage in restrictive eating behaviors, consuming some low-fat foods and largely avoiding high-fat foods (Alpers and Tuschen-Caffier, 2004; Bjorlie et al., 2022; Elran-Barak et al., 2015; Kales, 1990). We previously found that women with BN demonstrated these restrictive food choices on a food-related decision task, regardless of their affective state (Gianini et al., 2019). However, by leveraging a computational model that captures the decision-making process underlying ultimate choices, we found that negative affect more strongly biased individuals with BN than healthy adults, increasing the amount of time that taste information alone influenced their decisions involving food. This affect-induced delay in the consideration of healthiness versus tastiness may help account for the fact that negative affect is a common precursor to taste-driven overeating in healthy adults, and the fact that it is a particularly strong predictor of taste-driven, out-of-control eating in individuals with BN. Consistent with this notion, we found that negative affect-induced delays in accumulating health information relative to taste information for higher-fat foods were associated with more frequent out-of-control eating episodes in BN.

Previous studies have shown that in states of neutral affect, individuals with BN are more likely to consume foods that are low-fat and considered to be healthy (e.g., salads, fruits), as well as use healthiness information to guide their food choices (Alpers and Tuschen-Caffier, 2004; Bjorlie et al., 2022; Elran-Barak et al., 2015; Kales, 1990; Neveu et al., 2018; Schnepper et al., 2021). Our results demonstrate that in a neutral mood state, despite an initial tendency to focus on taste information before healthiness information, the BN group had stronger weights on healthiness information and lower, and in fact negatively signed, weights on taste, especially for high-fat foods. This negative weight on taste information (i.e., an aversive assignment to the value of taste) may have facilitated the BN group’s restrictive food choice policy compared to healthy controls. Individuals with BN may compensate for their tendency to focus on the tastiness of high-fat foods by negatively reappraising or deemphasizing the relative importance of taste and highly weighting their food choices based on health.

During binge-eating episodes, which often follow increases in negative affect, individuals with BN tend to consume large amounts of high-fat foods that were previously avoided (Alpers and Tuschen-Caffier, 2001; Berg et al., 2013; Collins et al., 2017; Haedt-Matt and Keel, 2011; Hilbert and Tuschen-Caffier, 2007; Smyth et al., 2007). Although prior studies examined food consumption associated with negative emotions in BN (Cardi et al., 2015), discrete food choice behaviors had not been investigated, and the influence of negative affect on specific aspects of the decision-making process was unclear. We showed that this affect-related change in eating behavior may occur because negative affect disrupts the delicate balance between how much weight tastiness and healthiness carry in influencing food choices and the relative speed with which they begin to exert their influence (Barakchian et al., 2021; HajiHosseini and Hutcherson, 2021; Hutcherson and Tusche, 2022; Lim et al., 2018; Maier et al., 2020; Schubert et al., 2021; Sullivan et al., 2015; Sullivan and Huettel, 2021). When in states of high negative affect, both healthy adults and individuals with BN may consider food’s appealing taste for longer, biasing them toward choosing tastier, high-fat foods. If this temporal advantage for tastiness is long enough, and a positive weight on tastiness is strong enough, individuals with BN could become more vulnerable to out-of-control eating because they could decide to eat tastier or high-fat foods before ever considering their perceived healthiness. This notion is consistent with our finding that greater affect-induced delays in processing healthiness information were associated with more frequent subjective binge episodes – episodes of dysregulated eating that may not include a large amount of food, but like objectively large binge-eating episodes, are dominated by carbohydrates and high-fat foods that are highly palatable (Presseller et al., 2023), and are often spurred by negative affect (Alpers and Tuschen-Caffier, 2001; Berg et al., 2013; Haedt-Matt and Keel, 2011; Hilbert and Tuschen-Caffier, 2007; Smyth et al., 2007).

Our findings reveal new insights into the dynamics of food-related decision-making in both healthy adults and BN and may help to inform new interventions. When attribute weights are inconsistent with a larger goal, decision-makers can use shifts in the timing of attribute consideration to achieve a desired outcome (Amasino et al., 2019; Maier et al., 2020). For example, earlier shifts in the relative onset time of healthiness information could help an individual with stronger weights on tastiness adhere to a diet. Perhaps targeting affect-induced shifts in the timing of attribute consideration could help regulate the over- and under-controlled eating seen in binge-fast cycles. For example, past research in individuals without eating disorders indicates that cueing attention toward the healthiness of foods during a choice task both increased the weight on healthiness information and increased its onset time during decision-making (Barakchian et al., 2021; Maier et al., 2020) but see Sullivan and Huettel, 2021. Several interventions already focus on modifying attentional biases in individuals with obesity and binge eating (Boutelle et al., 2016; Brockmeyer et al., 2019; Stojek et al., 2018). Our results suggest that such interventions could be targeted even more precisely. One way for individuals to avoid emotion-driven binge eating may be to focus attention away from the taste attributes of tastier, high-fat foods during states of high negative affect. Conversely, during states of low negative affect, increased attention on the positive taste attributes of all foods may be instrumental in motivating individuals with BN to engage in less restrictive eating.

**Figure 6.**
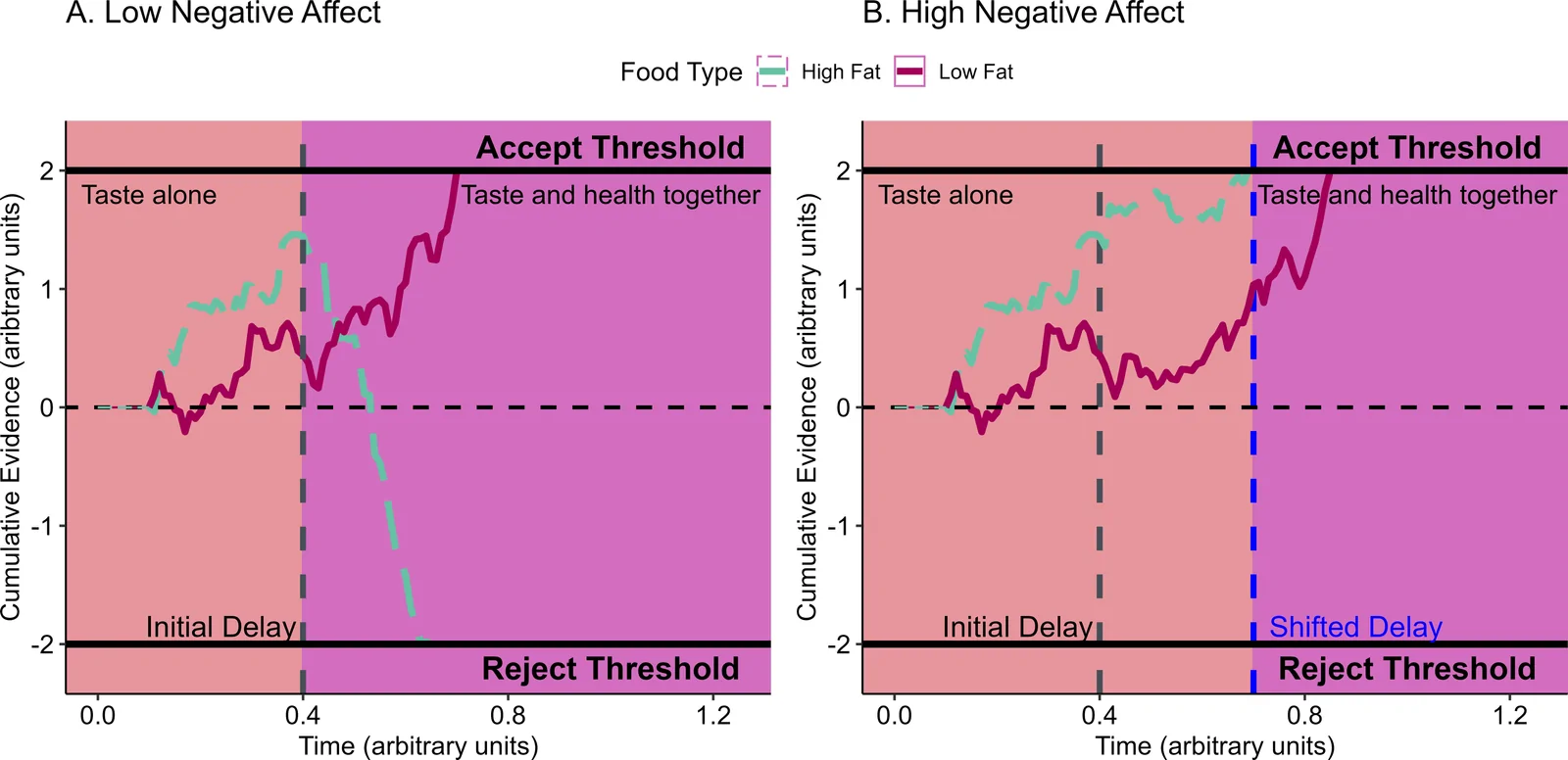
Revised model of affect-induced binge-eating. Each trajectory is a simulated individual with BN who considers the taste attribute alone (pink shaded sides of the panels) before considering both tastiness and healthiness attributes together (blue shaded sides of the panels). In both panels the solid purple trajectory illustrates a decision involving a low-fat food, where tastiness information has a weak, positive weight and healthiness information has a strong, positive weight. The dashed blue trajectory illustrates a decision involving a high-fat food, where tastiness information has a strong, positive weight and healthiness information has a strong, negative weight. (**A**) **Low negative affect**. During neutral affect, the high-fat food is trending toward the accept threshold until the onset of aversive healthiness information biases the trajectory toward the reject threshold. (**B**) **High negative affect**. During negative affect, the initial delay is shifted and tastiness information is accumulated longer before healthiness information comes online. With a longer delay in the onset of healthiness information, the evidence accumulation for the high-fat food has enough time to reach the accept threshold.

Although we found an aberrant influence of negative affect on the computations that contribute to food choice in BN, in our paradigm, the influence of negative affect was not strong enough to change final choices in either group. Even after the negative affect induction which decreased taste weights for HC and not BN, choice outcomes in BN were ultimately determined by the advantage in weighted evidence for healthiness as opposed to the advantage in relative timing for tastiness. We speculate that this may have been for two reasons. First, the affect induction method may not have been potent enough. In the current study, post-induction increases in negative affect (*d_HC_* = 1.34; *d_BN_* = 1.19) were smaller than those reported after other induction methods, such as viewing affectively salient images with congruent music (*d* = 3.33; Zhang et al., 2014), and they may not have been powerful enough to effect changes in ultimate choices. Although the Food Choice Task was completed immediately after the affect induction, we do not know how long its effect lasted because mood was not repeatedly assessed throughout the task. This and previous studies using task-based designs have only assessed mood ratings immediately following affect inductions (Rouhani et al., 2025; Werthmann et al., 2014), but future studies could include continuous assessments throughout the experiment to better quantify potential group differences in how induced affect changes persist and/or fluctuate. In addition, our negative-affect inducing music and writing exercise may have created an emotional experience that inadequately represents the negative affect that typically precedes emotional eating or binge eating. Qualitative studies of patients with BN indicate that interpersonal stressors and related negative emotions (e.g., resentment) often precipitate binge episodes (Bohon et al., 2021; Wasson, 2003). Perhaps larger increases in negative affect intensity or exposure to the specific types of negative affect which typically precede binge eating could produce a large enough asynchrony in onset timing of attributes and changes in attribute weights to change individuals’ choices (Figure 6). Second, participants knew they would be offered a snack-sized amount of food from a randomly selected trial to consume at the end of the task. This quantity of food may not have provided participants with access to the type of binge-eating experience that negative affect would otherwise precipitate. Further work is needed to test whether the same processes identified in the current study also drive binge eating outside the lab (Wonderlich et al., 2022). Future adjustments to incentive compatibility, with designs that allow for increased consumption beyond snack-sized portions and/or guaranteed access to a wider-array of high-fat foods could clarify whether opportunities to overconsume highly palatable food would generate different patterns of food choice behavior on the task. Additionally, future research should include continuous assessments of other relevant factors such as cravings (Konova et al., 2018; Wonderlich et al., 2017) to enrich the connection between mood, discrete food choice, and binge eating.

Of note, the modest sample size (*n* = 46) composed exclusively of female participants may limit the generalizability of our findings. Replication in larger samples that include male participants is needed. Nonetheless, the study’s repeated-measures design benefits from the added statistical power and sensitivity of within-subjects statistical tests, which reduce error variance by controlling for individual differences (Charness et al., 2012; Judd et al., 2017; Westfall et al., 2014). Moreover, we collected a large quantity of data per subject, which enhances the validity of the findings by providing a more precise estimate of the effects of the experimental manipulation, despite the smaller sample size (Baker et al., 2021; Smith and Little, 2018).

Overall, our results advance our understanding of how changes in affective states may shift individuals with BN away from typically restrictive dietary choices and toward binge eating. Specifically, negative affect can bias individuals with BN to focus on taste information for longer, and the strength of this effect for high-fat foods was associated with symptom severity. Our results add to recent data suggesting that computational modeling can detect subtle alterations in decision-making processes in BN that are sensitive to state changes (Berner et al., 2023), and future research should continue examining the potential connections between negative affect and eating-related decision-making using these analytic approaches.

## Materials and methods

### Participants

Data were collected from 25 individuals who met *DSM-5* diagnostic criteria for BN and 21 healthy controls, group-matched for age and BMI. All participants were female and aged 18–40 years. The sample consisted of 27 participants who identified as Caucasian (58.7%), 7 as Hispanic (15.2%), 6 as Asian (13.0%), 3 as Black (6.5%), and 3 as mixed race (6.5%). For details on recruitment and exclusions, see Gianini et al., 2019.

### Materials and procedures

Participants completed the tasks over two separate study sessions, each lasting approximately 4 hr. On each study day, participants were instructed to abstain from consuming food or drinks except for a standardized meal consumed 2 hr preceding the study session.

Participants completed a Food Choice Task (Steinglass et al., 2015) on two separate days, in two counterbalanced states: after a neutral affect induction, and after a negative affect induction. This task is composed of three phases: Healthiness Rating, Tastiness Rating, and Choice. In each phase, participants viewed the same 43 food items, each categorized as either low-fat (<30% kcals from fat) or high-fat (>30% kcals from fat). During the Health Rating phase, participants used a 5-point scale to rate the healthiness of each food item from 1 (Unhealthy) to 3 (Neutral) to 5 (Healthy). During the Taste Rating phase, participants used a 5-point scale to rate the tastiness of each food item from 1 (Bad) to 3 (Neutral) to 5 (Good). The order of the Health Rating and Taste Rating phases was counterbalanced. After both phases were completed, one food item that was rated Neutral for both health and taste was selected as a Reference Item for use in the Choice phase. If no item was rated Neutral for healthiness and tastiness, then a food item rated 3 on health and 4 or above on taste was selected following previously established procedures (Steinglass et al., 2015). During the Choice phase, participants made choices between the neutral Reference Item and other food items presented on the computer screen. Participants indicated their choice on a 5-point scale from 1 (No – select the Reference Item) to 3 (Indifference) to 5 (Yes – select the shown food item). Participants were informed that one randomly selected trial would be selected for payout at the end of the experiment and would receive the food item they selected on that trial.

The experiment included an affect induction that included a combination of music and autobiographical writing (Werthmann et al., 2014). During the negative affect induction, participants were asked to write about a recent negative experience while listening to ‘Adagio for Strings’ by Samuel Barber for 8 min. During the neutral affect induction, participants were asked to write about the route they took to get to the study site while listening to ‘Dancing with the Sun’ by Celia Felix for 8 min. The 65-item Profile of Mood States scale was administered before and after the affect inductions to assess changes in affect (McNair et al., 1971).

Participants also completed the following measures: Eating Disorder Examination Questionnaire (EDE-Q; Fairburn and Beglin, 1994); Difficulties in Emotion Regulation Scale (DERS; Gratz and Roemer, 2004); Urgency, Premeditation, Perseveration, Sensation Seeking, and Positive Urgency Behavior Scale-Negative Urgency subscale (UPPS-P Negative Urgency; Lynam et al., 2006); Beck Depression Inventory (BDI; Beck et al., 1961); State Anxiety Inventory (STAI; Spielberger et al., 1983).

For additional information, please see Gianini et al., 2019.

### Data analysis

#### Data transformations and exclusions

Following the procedure from Gianini et al., 2019, participants’ responses from the Choice phase of the FCT were binarized from their 5-point scale. Responses marked 1 or 2 (i.e., ‘no’) were determined to be choices of the reference item, responses marked 4 or 5 (i.e., ‘yes’) were determined to be choices of the presented food item, and responses marked 3 (i.e., ‘indifferent’) were omitted (total *n* = 599; BN *n* = 292, HC *n* = 307).

In contrast to the original study, we excluded trials containing outlier response times (RTs). Outlier response times likely reflect phenomena outside the decision-making processes of interest, including attention lapses or accidental responses (Cousineau and Chartier, 2010; Ratcliff, 1993). We excluded outlier responses using cutoffs of ±3 SDs from the mean (Berger and Kiefer, 2021). For each participant, the mean and standard deviation of their RTs were calculated and trials containing responses larger/smaller than the mean ± 3 SDs were excluded. Additionally, we removed trials where the RTs were greater than 10,000 ms or less than 250 ms after the mean ± 3 SDs treatment. Using this method, we removed 2.5% of trials (total *n* = 85; BN *n* = 51, HC *n* = 34).

#### Computational modeling

We used a time-varying DDM (Ratcliff and McKoon, 2008) to study the dynamics of the decision-making during the Food Choice Task. This time-varying model introduces a starting time parameter that indicates the delay with which different attributes enter the evidence accumulation process. This model was previously fitted to food choice data (Lombardi and Hare, 2021; Maier et al., 2020) and lottery choice data (Chen et al., 2022) from healthy adults.

The stDDM was estimated using a hierarchical, Bayesian framework implemented with the R package *Rjags*, which uses the JAGS MCMC sampling algorithm (Plummer, 2003). Our fitting scripts were adapted from those developed by Hsiang-Yu Chen, Gaia Lombardo, and Todd Hare (Chen et al., 2022). This fitting method simultaneously estimates both group- and individual-level parameters, which improves the reliability of parameters estimated from data with low trial counts per participant (Ratcliff and Childers, 2015; Wiecki et al., 2013). Parameter estimates were fitted to data that included individual-level response times, choices, *z*-scored health and taste ratings, and Affect Condition. We specified a model where individual parameters were drawn from four separate group-level distributions that varied both by Group (BN or HC) and by Affect Induction (neutral or negative). Within this model, attribute weight parameters $\left (\omega _{Taste}\,\mathrm{and} \,\omega _{Health}\right)$ and the relative-starting time parameter $\left (\tau _{s}\right)$ were estimated separately based on food-types (low-fat or high-fat). Using model comparisons and posterior predictive checks, we confirmed that this model best fit our data compared to simpler models (see Appendix 1).

Following previously described protocols (Chen et al., 2022), we used group-level priors for attribute weight parameters $\left (\omega _{Taste}\,\mathrm{and} \,\omega _{Health}\right)$, the relative-starting time parameter $\left (\tau _{s}\right)$, and the affect induction parameters that were drawn from Gaussian distributions with mean = 0 and SD = 1. These parameter values were then divided by their standard deviations before fitting the model. The priors for boundary separation $\left (\alpha \right)$ and non-decision time $\left (\tau _{ND}\right)$ were drawn from uniform distributions with ranges 1.0 × 10^−4^ to 5 and 0–10, respectively. The priors for starting point bias $\left (z\right)$ were drawn from a beta distribution where both the shape and scale parameters were set to 2. All individual-level priors were drawn from gamma distributions with shape parameters of 1 and scale parameters of 0.1. Posterior estimates were drawn from three chains, each with 100,000 samples (85,000 discarded as burn-in) and thinning every 10 samples. Convergence among chains was assessed using the Gelman-Rubin statistic (Gelman and Rubin, 1992) using a threshold of 1.1. Model fit was quantified with the Widely Applicable Information Criterion (WAIC) (Watanabe, 2010), which penalizes for model complexity based on the number of parameters to prevent overfitting. For parametric tests involving parameter estimates, we used the medians of the individual-level posterior distributions. See the Appendix 1 for model information on model comparisons, model identifiability, posterior predictive checks, and parameter recovery.

Multilevel linear regressions were run to evaluate our hypotheses regarding the influence of Food Type, affect induction, BN diagnosis, and their interactions on parameter estimates. Each regression model included Food Type (coded –1/1 for low-fat/high-fat foods), Affect Condition (coded –1/1 for Neutral/Negative), and Group (coded –1/1 for HC/BN), and their interactions as independent variables. For these models, the intercepts were treated as random effects and statistical significance was evaluated using α=0.050.

Because the parameters of interest (i.e., *τ_s_*, *ω_Taste_*, and *ω_Health_*) were estimated separately per condition and food type, each participant contributed exactly four observations to these models (2 affect conditions × 2 food types), creating a fully balanced but structured repeated-measures design. Standard random-intercept-only models implemented in *lme4* (Bates et al., 2015) and *lmerTest* (Kuznetsova et al., 2013) do not explicitly model the residual covariance among these four within-person observations. To address this, models were re-implemented using the *nlme* package (Pinheiro et al., 2022), which permits explicit specification of the within-subject residual covariance structure.

We evaluated three candidate covariance structures. We first considered a nested random effect of affect condition within subject, which would directly encode the pairing of Low-Fat and High-Fat observations within each session, but this model failed to converge due to the limit. With only two observations per session per subject, the session-level and residual variance components cannot be separately estimated. Next, we implemented a compound symmetry structure using the *corCompSymm* function, which assumes equal correlations among all four within-person observations. Finally, we implemented an unstructured covariance matrix using the *corSymm* function, which places no constraints on the pattern of correlations among the four within-person observations and represents the most general feasible specification for this design. Model comparisons via likelihood ratio test and information criteria (LRT: Δdf = 5, p = 0.057; AIC (−119.08 vs. –118.34) marginally favored unstructured; BIC (−57.99 vs. –73.33) favored compound symmetry) did not strongly favor either tractable structure, and we selected the unstructured model as the more conservative option, as all constrained covariance structures are nested within the unstructured model and represent testable restrictions on the covariance parameters (Molenberghs and Verbeke, 2000; Pinheiro and Bates, 2000). All models additionally included heterogeneous residual variances across the four condition-by-food-type cells using the *varIdent* function, allowing each cell to have its own residual standard deviation.

Difference-in-differences scores were additionally computed for each participant and parameter to provide a complementary analysis that encodes the within-session pairing directly by construction, bypassing the covariance specification problem entirely. These analyses and their results are described in the Appendix 1.

To unpack observed interaction effects, we ran exploratory simple effects analyses to separately evaluate the influence of independent variables at specific values of other independent variables (Aiken et al., 1991; Brambor et al., 2006; Jaccard and Turrisi, 2003; Winer et al., 1991). We used the R package *emmeans* to compute estimated marginal means for specified factor combinations (Lenth, 2025), evaluating statistical significance using α=0.050.

Additional fixed-effects regressions were run to assess the relationship between parameter estimates and symptom severity. For these exploratory analyses, we used negative binomial models to test the association between parameter estimates and the 3-month frequency of retrospectively reported OBEs and SBEs. We implemented these models using the R package *glmmTMB* (Brooks et al., 2017), with a zero-inflated term included for the model predicting SBE. For each of these two models, individual-level parameter estimates from each condition and food-type were included as independent variables. We applied a Bonferroni correction to control for the FWE of these exploratory analyses, using α=0.025.

### Code availability

Custom code in this work is available at https://github.com/blairshevlin/Computations_BN_Food_Choice, copy archived at Shevlin, 2026.

## Data Availability

The data supporting the findings of this study are not publicly available because participants did not provide consent for their data to be shared. De-identified data may be requested by contacting Joanna Steinglass (js1124@cumc.columbia.edu). There is no standing formal application process; each request will be considered on a case-by-case basis by the study team to confirm that the proposed use is consistent with the consent provided by study participants. Requesters must also have appropriate ethical/IRB approval from their own institution prior to any data release. This applies equally to academic and non-academic (e.g., commercial) requests, as each request will be evaluated individually against these criteria. The code for the analyses presented in this paper is openly accessible at https://github.com/blairshevlin/Computations_BN_Food_Choice, copy archived at Shevlin, 2026.

## Additional files

MDAR checklist

## Appendix 1

### Supplementary methods and results

#### Supplementary methods

##### Behavioral analyses

We used a similar analytic approach to assessing choice and response time data to the methods described in Gianini et al., 2019. A multilevel logistic regression model was fit to trial-level choice data (coded 1/0 for yes/no) using Food Type (coded –1/1 for low-fat/high-fat foods), Affect Condition (coded –1/1 for Neutral/Negative), Group (coded –1/1 for HC/BN), and their interactions as independent variables. We fit a second logistic regression model to trial-level choice data, also using Affect Condition, Group, and their interaction as independent variables. In this second model, we also included *z*-scored health and taste ratings, and their independent interactions with group, as independent variables.

We assessed self-control by selecting trials where participants made decisions involving highly appetitive unhealthy foods (i.e., taste rating = 5, health rating = 1) or unappetizing healthy foods (i.e., taste rating = 1, health rating = 5). The use of self-control (coded 1 for rejecting appetitive unhealthy foods or selecting unappetizing healthy foods and 0 otherwise) was assessed using a multilevel logistic regression with Affect Condition, Group, and their interaction entered as independent variables.

A similar multilevel linear regression was fit to trial-level response time data (log-transformed) using the same independent variables. This model tested the main effects and interactions of Food Type, Affect Condition, and Group with a dummy choice variable (coded –1/1 for choice of reference item or food item).

For all models, any within-individual variables (i.e., the intercept, main effects of z-scored health and taste ratings, Affect Condition, and Food Type, and their interactions) were treated as random effects. Regression analyses were conducted using the R packages *lme4* (Bates et al., 2015) and *nmle* (Pinheiro et al., 2022) and marginal means were estimated using *emmeans* (Lenth et al., 2024) and *sjPlot* (Lüdecke et al., 2024).

##### Model comparison

We assessed the model fit of variants of the time-varying DDM to determine which model parameters best explained the influence of Food Type on behavior. The null model (M0) assumed decision parameters were invariant toward food type. The attribute weight model (M1) assumed both $\omega _{taste}$ and $\omega _{health}$ varied based on food type. The attribute starting time model (M2) assumed $\tau _{s}$ varied based on food type. Finally, the full model (M3) assumed $\omega _{taste}$, $\omega _{health}$, and $\tau _{s}$ varied based on food type. All models were estimated using the same procedures described in the Computational model subsection of the Methods section located in the main text.

##### Model identifiability and posterior predictive checks

We then evaluated the identifiability of each model. For each model, a set of simulated datasets was generated reflecting the structure and parameters of the corresponding model. First, each model was fit to the empirical data using the procedures described in the Materials and Methods section of the main text, under the subsection *Computational modeling*. Data were simulated using the empirical trial structure and the medians of the individual-level posterior parameter distributions. Each simulated dataset was fit using all four models (M0, M1, M2, and M3), regardless of the generating model. For model fitting, we employed the same Bayesian hierarchical framework that was used in previous steps. We evaluated model identifiability by determining the frequency with which the model that generated the data was also identified as the best-fitting model, based on WAIC (Watanabe, 2010) implemented in the *loo* package in R (Vehtari et al., 2017; Vehtari et al., 2024). This allowed us to assess the extent to which each model was uniquely identifiable and distinguishable from alternative models. A model was considered successfully recovered when it was the best-fitting model for the data it generated.

In addition, we performed posterior predictive checks to ensure that the models could capture key features of the observed data. We assessed the simulated choice and response time patterns of the model variants using nonparametric equivalent aligned rank tests (Wobbrock et al., 2011) implemented in the *ARTool* package in R (Kay et al., 2021). For this procedure, we calculated both the empirical and the simulated mean choice proportions and median response times for each participant across Affect Conditions and Food Types. Aligned rank tests were run separately on mean choice proportions and median response times evaluating the interaction between Group, Affect Condition, Food Type, and Source (empirical or simulated data) for each model. We subsequently ran the multi-level logistic regression models predicting trial-level choice from the interaction between Group, Affect Condition, and Food Type on the simulated data to confirm we could recover the results from the empirical data.

##### Parameter recovery

We conducted a parameter recoverability exercise to ensure that the values of the winning model could be accurately recovered from data that had similar qualities to the empirical data included in the current study. For this exercise, we took the mean values of the subject-level parameters (*χ*_fit) estimated in both the neutral and negative affect conditions and simulated data using the trial-level health and taste ratings from the empirical data used during estimation. This synthetic data was then used to fit the winning model using the procedures described previously. We extracted this new set of parameters, once again calculating the mean values of the subject-level parameters (*χ*_rec). We ran Pearson correlations between *χ*_fit and *χ*_rec to assess parameter recovery.

##### Analysis of additional DDM parameters

Mixed-effects regression models were fit to non-decision time ($\tau _{ND}$), boundary separation (α), and starting point bias (z), following the same estimation procedures described in the main text. As these parameters did not include food type as a factor, each participant contributed two observations (one per affect condition). Where significant or trending group by condition interactions were observed, condition effect scores (negative minus neutral) were compared between groups using two-sample Wilcoxon rank-sum tests, with additional one-sample Wilcoxon signed-rank tests run within each group to assess whether the condition effect differed from zero.

##### Confirmatory analysis of DDM parameters

To confirm the presence of significant interactions identified in the mixed-effects regression models, we conducted difference-in-difference analyses for each DDM parameter where a significant two-way or three-way interaction was observed. For each participant, we computed two sets of difference scores. For parameters with a significant three-way interaction (Group × Condition × Food Type), we calculated the food-type effect within each condition (high-fat minus low-fat) separately for the neutral and negative affect conditions, the condition effect within each food type (negative minus neutral) separately for low-fat and high-fat foods, and an overall difference-in-difference score by subtracting the food-type effect in the neutral condition from the food-type effect in the negative condition. For parameters with a significant two-way interaction, we computed the relevant difference score corresponding to the interaction of interest. Group differences in each of these scores were assessed using Wilcoxon rank-sum tests.

##### Mood analysis

We analyzed the specific emotions that changed following the mood inductions. As described in the main text, mood was assessed using the Profile of Mood States (POMS; McNair et al., 1971). The POMS has the following subscales: Anger, Confusion, Depression, Fatigue, Tension, and Vigor.

##### Mood induction effectiveness

To assess the effectiveness of the mood induction in the BN group, we conducted follow-up simple effects analyses using estimated marginal means from our primary linear mixed-effects model (reported in Appendix 1—table 1). This model included group (HC = 0, BN = 1), condition (Neutral = 0, Negative = 1), and time (pre-induction=0, post-induction=1) as fixed effects, all interactions, and random intercepts for participants to account for within-subject correlations.

We conducted two complementary analyses. First, we examined the group × time interaction within each condition separately to assess whether the mood induction produced significant changes in negative affect within the BN group. Second, we directly compared post-induction affect between conditions within the BN group to determine whether participants experienced meaningfully different affective states when completing the food decision task.

Effect sizes for within-subject comparisons were calculated as Cohen’s d using the mean difference divided by the pooled standard deviation of the repeated measures.

##### Alternative symptom severity measure

Recent studies suggest that negative urgency (i.e., acting impulsively when experiencing negative affect) contributes to binge-eating pathology (Wonderlich et al., 2024). Given the restrictive food choice behavior we observed in this study, decision parameters might correlate with symptom severity measures related to restrictive behaviors outside the lab. We used linear regressions to evaluate the extent to which subject-level parameters (i.e., $\tau _{s}$, $\omega _{taste}$, $\omega _{health}$) could predict scores on the negative urgency subscale of the UPPS-P Impulsive Behavior Scale (Lynam et al., 2006) and the restraint subscale of the Eating Disorder Examination Questionnaire (EDE-Q; Fairburn and Beglin, 1994).

##### Association between dimensions of symptom severity

To address concerns about the specificity of associations with binge eating versus other eating disorder symptoms, we examined the correlation between self-reported binge frequency and dietary restraint in our BN sample. Specifically, we assessed the relationships between the EDE-Q Restraint subscale and two measures of binge frequency over the past 3 months: subjective binge episodes (SBEs; episodes involving a loss of control over eating but without consuming an objectively large amount of food) and objective binge episodes (OBEs; episodes involving both loss of control and consumption of an objectively large amount of food). Given that binge frequency data are typically positively skewed (i.e., log-normally distributed), we used Spearman’s rank correlation (*ρ*) to examine these associations, as this non-parametric approach is robust to violations of normality assumptions.

### Supplementary results

#### Analysis of choice patterns

We replicated the findings from the original analysis, confirming that individuals with BN were more likely than the HC group to choose the reference item (Appendix 1—table 9; $\beta =- 0.48,z=- 2.63,\rm p=0.009$), with the BN group selecting the reference item in approximately 68.3% of trials (95% CI: 57.0%–77.8%) and the HC group selecting it in 45.3% of trials (95% CI: 33.9%–58.3%). We also replicated the Group-by-Food Type interaction reported in the original analysis $\left (\beta =- 0.27,z=- 1.99,\rm p=0.047\right)$. Those in the HC group chose approximately 63.1% (95% CI: 52.0%–73.0%) of low-fat foods and 46.0% (95% CI: 27.8%–65.3%) of high-fat foods, while individuals with BN chose 53.0% (95% CI: 42.6%–63.1%) of low-fat foods and 16.1% (95% CI: 8.3%–28.8%) of high-fat foods. While individuals with BN were less likely to choose the presented foods over the neutral reference item in general, both groups chose high-fat foods less often than low-fat foods $\left (\beta =- 0.62,z=- 4.58,p=4.75\times 10^{- 6}\right)$.

Similar to the original analysis, we did not observe a significant main effect of Affect Condition $\left (\beta =- 0.02,z=- 0.26,\rm p=0.793\right)$, an interaction between Affect Condition and Group $\left (\beta =0.07,z=0.82,\rm p=0.410\right)$, or an interaction between Affect Condition and Food Type $\left (\beta =- 0.03,z=- 0.62,\rm p=0.535\right)$ on food choice. We also did not observe a significant three-way interaction between Affect Condition, Food Type, and Group on choice $\left (\beta =- 0.10,z=- 1.91,\rm p=0.056\right)$.

In a separate multilevel regression (Appendix 1—table 10), we examined the relationships among food choice, taste, and health ratings, BN diagnosis, and negative affect. We confirmed the original paper’s finding that health ratings more strongly influenced food choice among the BN group compared to the HC group $\left (\beta =0.64,z=2.77,\rm p=0.006\right)$. We additionally observed that taste ratings were less influential among the BN group compared to the HC group $\left (\beta =- 0.65,z=- 2.78,\rm p=0.005\right)$. We also reproduced the original paper’s finding of a significant, negative interaction between Affect Condition and taste ratings on choice, such that negative affect reduced the influence of taste ratings on choice among both groups $\left (\beta =- 0.45,z=- 2.57,\rm p=0.010\right)$.

Furthermore, we examined trials with an opportunity for self-control (Appendix 1—table 11). Reproducing the original analyses, we found that the BN group used self-control more often than the HC group $\left (\beta =0.38,z=2.14,\rm p=0.033\right)$. We did not observe a main effect of Affect Condition on self-control $\left (\beta =0.08,z=0.70,\rm p=0.486\right)$, nor an interaction between Affect Condition and Group $\left (\beta =0.15,z=1.27,\rm p=0.204\right)$.

We next examined RT patterns using multilevel regression models fit to trial-level RT data (Appendix 1—table 12). We did not observe significant main effects of Affect Condition or its interactions with other variables. An interaction between Food Type and choice on RT indicated that, across groups, rejections were relatively faster for high-fat foods than for low-fat foods, while selections were relatively slower for high-fat foods than for low-fat foods $\left (\beta =0.03,t=2.70,\rm p=0.007\right)$. Additionally, we found a positive interaction between Group and choice, suggesting that the BN group was slower than the HC group to choose presented foods over the neutral reference item $\left (\beta =0.03,t=2.44,\rm p=0.015\right)$.

#### Modeling results

##### Model comparison

These different model variants are described in Appendix 1—table 13. The winning model (M3) included terms representing the influence of Food Type on both attribute weights ($\omega _{taste}$, $\omega _{health}$) and attribute starting time ($\tau _{s}$).

##### Model identifiability

The results of the model recovery exercise are summarized in Appendix 1—table 14. Models M0, M1, and M3 all had perfect recoverability. However, M2 had poor recoverability, as it was always misidentified as M3.

##### Posterior predictive check

The data from M3 best replicated the results from the empirical data. While all models had biases in predicting choice data, these were smallest in M3. M0 (Appendix 1—table 15) was significantly biased in predicting choice based on Food Type ($F\left (1,352\right)=11.97,\rm p=0.001$) and was biased in predicting response times ($F\left (1,352\right)=7.88,\rm p=0.005$). Both M1 (Appendix 1—table 16) and M2 (Appendix 1—table 17) were similarly biased in predicting choice based on Food Type (M1: $F\left (1,352\right)=45.07,p=7.63\times 10^{- 11}$; M2: $F\left (1,352\right)=4.90,\rm p=0.028$), but M3 (Appendix 1—table 18) was not ($F\left (1,352\right)=1.70,\rm p=0.193$).

In the empirical data, a multilevel logistic regression found significant, negative main effects of Group and Food Type, and a negative Group × Food Type interaction (Appendix 1—table 9). M0 (Appendix 1—table 19) was able to reproduce the main effect of Group ($\beta =- 0.34,t=- 2.74,\rm p=0.006$) and the Group × Food Type interaction ($\beta =- 0.32,t=- 4.34,p=1.41\times 10^{- 5}$), but not the main effect of Food Type ($\beta =- 0.01,t=- 0.19,\rm p=0.848$). M1 (Appendix 1—table 20) produced a marginal negative effect of Group ($\beta =- 0.28,t=- 1.93,\rm p=0.053$) and the negative Group × Food Type interaction ($\beta =- 0.22,t=- 3.27,\rm p=0.001$), but the significant effect of Food Type had the wrong sign ($\beta =0.42,t=6.35,p=2.17\times 10^{- 10}$). M2 (Appendix 1—table 21) produced the negative Group effect ($\beta =- 0.42,t=- 3.08,\rm p=0.002$) and the negative Group × Food Type interaction ($\beta =- 0.36,t=- 3.36,p=7.93\times 10^{- 4}$), but not the significant effect of Food Type ($\beta =- 0.16,t=- 1.52,\rm p=0.128$). Only M3 (Appendix 1—table 22) could reproduce all three patterns: a negative effect of Group ($\beta =- 0.36,t=- 2.68,\rm p=0.007$), a negative effect of Food Type ($\beta =- 0.36,t=- 4.16,p=3.25\times 10^{- 5}$), and a negative interaction between the two ($\beta =- 0.27,t=- 3.11,\rm p=0.002$).

##### Parameter recovery

As illustrated in Figure 4—figure supplement 1, recovery was satisfactory for all parameters of M3.

#### Analysis of additional DDM parameters

##### Non-decision time

We did not observe significant effects of either Group or Affect Condition in non-decision time ($\tau _{ND}$; Appendix 1—table 23). However, we found a significant Group by Affect Condition interaction (*β* = –0.091, *t* = –2.19, p = 0.034), suggesting that the BN group, compared to the HC group, had faster non-decision times following the negative affect induction versus the neutral condition. Although the two-sample Wilcoxon test comparing the condition effect between groups fell just short of significance (*W* = 351, p = 0.058), one-sample tests indicated that the condition effect was non-significant in HC (*W* = 143, p = 0.355) while trending toward significance in BN (*W* = 96, p = 0.075), consistent with a selective speeding of non-decision time under negative affect in BN that was not observed in HC.

##### Boundary separation

For the boundary separation parameter (*α*; Appendix 1—table 24), there was a significant difference between the HC and BN groups (*β* = –0.397, *t* = –2.84, p = 0.007), with the BN group exhibiting reduced response caution compared to the HC group. We found a significant negative effect of Affect Condition (*β* = –0.366, *t* = –4.26, p < 0.001), and a trending positive interaction between Group and Affect Condition (*β* = 0.227, *t* = 1.94, p = 0.058). One-sample Wilcoxon tests confirmed that the negative affect induction significantly reduced boundary separation in HC (*V* = 13, p < 0.001), but not in BN (*V* = 99, p = 0.090), though the group difference in condition effect fell just short of significance (*W* = 187, p = 0.098). Together, these results suggest that negative affect prompted greater response caution reductions in HC than in BN, consistent with the trending interaction, though the non-significant Wilcoxon two-sample test urges caution in this interpretation.

##### Starting point bias

There were no Group or Affect Condition differences in starting point bias (*z*; Appendix 1—table 25).

#### Confirmatory analysis of DDM parameters

##### Relative attribute onset ($\tau _{s}$)

To confirm the significant three-way interaction (Group × Condition × Food Type), we computed difference-in-difference scores for each participant. In the neutral condition, HC showed a larger food-type effect than BN (HC: *M* = 0.14, BN: *M* = –0.15; *W* = 367, p = 0.021), while this group difference was absent in the negative affect condition (HC: *M* = 0.02, BN: *M* = 0.00; *W* = 270, p = 0.879). Groups also differed in how negative affect modulated attribute onset for low-fat foods (HC: *M* = –0.14, BN: *M* = –0.31; *W* = 359, p = 0.033) but not high-fat foods (HC: *M* = –0.20, BN: *M* = –0.16; *W* = 280, p = 0.710). The overall difference-in-difference score was significantly larger in BN than HC (HC: *M* = –0.10, BN: *M* = 0.15; *W* = 156, p = 0.018), confirming the presence of the three-way interaction.

##### Taste weights ($\omega _{taste}$)

To confirm the significant two-way interaction between group and condition, we compared groups on the condition effect within each food type. Groups differed significantly in the condition effect for low-fat foods (HC: *M* = –0.16, BN: *M* = 0.02; *W* = 162, p = 0.026) but not high-fat foods (HC: *M* = –0.13, BN: *M* = –0.07; *W* = 221, p = 0.369), confirming that the group difference in response to negative affect induction was specific to low-fat foods. To confirm the significant two-way interaction between group and food type in the negative affect condition, groups did not differ in the food-type effect in the neutral condition (HC: *M* = –0.24, BN: *M* = –0.29; *W* = 292, p = 0.526), but did differ significantly in the negative affect condition (HC: *M* = –0.22, BN: *M* = –0.38; *W* = 372, p = 0.015), confirming that the group difference in food-type bias emerged specifically following negative affect induction.

##### Healthiness weights ($\omega _{health}$)

To confirm the significant two-way interaction between group and food type, we compared groups on the food-type effect within each condition. Groups differed significantly in both the neutral condition (HC: *M* = –0.04, BN: *M* = 0.24; *W* = 133, p = 0.004) and the negative affect condition (HC: *M* = 0.03, BN: *M* = 0.27; *W* = 156, p = 0.018), confirming that BN placed consistently greater weight on health information for high-fat versus low-fat foods regardless of affective state, while HC did not.

#### Mood analysis

##### Anger subscale

We found that the BN group had significantly higher scores on the Anger subscale (Appendix 1—table 26; $\beta =7.59,t=3.16,\rm p=0.002$). We also found a significant, positive interaction between Affect Condition and Timing ($\beta =5.71,t=3.04,\rm p=0.003$), indicating that the negative affect induction increased anger ratings across Groups.

##### Confusion subscale

We found that the BN group had significantly higher scores on the Confusion subscale (Appendix 1—table 27; $\beta =6.07,t=4.75,p=1.03\times 10^{- 5}$). We also found a significant, positive interaction between Affect Condition and Timing ($\beta =2.30,t=2.42,\rm p=0.017$), indicating that the negative affect induction increased confusion ratings across Groups.

##### Depression subscale

We found that the BN group had significantly higher scores on the Depression subscale (Appendix 1—table 28; $\beta =16.06,t=4.63,p=1.84\times 10^{- 5}$). We also found a significant, positive interaction between Affect Condition and Timing (β=5.58,t=2.44,p=0.016), indicating that the negative affect induction increased depression ratings across Groups.

##### Fatigue subscale

We found that the BN group had significantly higher scores on the Fatigue subscale (Appendix 1—table 29; β=6.04,t=3.34,p=0.001). No other significant contrasts emerged.

##### Tension subscale

We found that the BN group had significantly higher scores on the Tension subscale (Appendix 1—table 30; $\beta =8.76,t=4.38,p=5.05\times 10^{- 5}$). We also found both a significant, negative interaction between Group and Timing ($\beta =- 2.50,t=1.67,\rm p=0.021$) and a significant, positive interaction between Group, Affect Condition, and Timing ($\beta =3.82,t=2.53,\rm p=0.013$). Taken together, these indicate that only the BN group’s tension ratings changed after the affect induction, but in a condition-dependent way: decreasing tension following the neutral affect induction and increasing tension following the negative affect induction.

##### Vigor subscale

We found that the BN group had significantly lower scores on the Vigor subscale (Appendix 1—table 31; $\beta =- 10.40,t=- 5.73,p=2.33\times 10^{- 7}$). We also found a significant, negative interaction between Affect Condition and Timing ($\beta =- 4.93,t=- 3.58,\rm p=0.001$), indicating that the negative affect induction decreased vigor ratings across Groups.

#### Effectiveness of mood induction in the BN group

In the first analysis, we examined changes in negative affect from pre- to post-induction within each condition for the BN group. In the Negative condition, individuals with bulimia nervosa demonstrated a substantial increase in negative affect from pre- to post-induction (mean difference = 20.36, SE = 4.21, *t* = 4.84, p < 0.0001, Cohen’s *d* = 0.97). This large effect size indicates that the negative mood induction produced a meaningful increase in negative affect. In contrast, the Neutral condition showed no significant change in negative affect in the BN group (mean difference = 7.16, SE = 4.21, *t* = 1.70, p = 0.327, Cohen’s *d* = 0.34).

In the second analysis, we directly compared post-induction negative affect between conditions within the BN group. Reported negative affect was significantly higher following the negative mood induction than after the neutral mood induction (mean difference = 17.40, SE = 4.21, *t* = 4.13, p = 0.0003, Cohen’s *d* = 0.83). This large effect size represents a meaningful and statistically robust difference in affective states between conditions.

These within-group effects confirm that the negative mood induction was effective in producing increased negative affect in the BN group and resulted in significantly different affective states between the negative and neutral conditions. Critically, these findings demonstrate that BN participants completed the food decision task under meaningfully different affective states in the two conditions, supporting the interpretability of the subsequent analyses.

##### Alternative symptom severity measures

There were no significant relationships between negative urgency ratings, decision parameters (Appendix 1—table 32), or restrictive behaviors (Appendix 1—table 33).

##### Associations between symptom severity measures

The correlation between EDE-Q Restraint and subjective binge episodes was small and non-significant (*ρ* = 0.21, *S* = 2045.2, p = 0.306). Similarly, the correlation between EDE-Q Restraint and objective binge episodes was near zero and non-significant (*ρ* = 0.05, *S* = 2465.7, p = 0.806). These results indicate that subjective binge frequency and dietary restraint were relatively independent dimensions of eating pathology in our sample. This dissociation supports the specificity of our primary findings: the fact that our DDM parameters were associated with binge frequency but not with dietary restraint suggests that the affect-induced changes in decision-making we observed are specifically related to loss-of-control eating behavior rather than reflecting a general correlate of dietary restraint.

**Appendix 1—table 1.** Linear mixed-effects regression analyzing overall negative affect across Group, Affect Condition, and Timing.

Predictors		*B*	SE	*t*	p
Intercept		–7.14	7.35	–0.97	0.335
Group		55.02	9.97	5.52	**<0.001**
Affect Condition		–2.43	4.49	–0.54	0.590
Timing		0.05	4.49	0.01	0.992
Group × Affect Condition		–7.69	6.09	–1.26	0.209
Group × Timing		–7.21	6.09	–1.18	0.239
Affect Condition × Timing		20.43	6.35	3.22	**0.002**
Group × Affect Condition × Timing		7.09	8.62	0.82	0.412
*N*_subject_	46				
Observations	184				

Bold p-values indicate p < 0.05.

**Appendix 1—table 2.** Linear mixed-effects regression analyzing attribute onset ($\tau _{s}$) across Group, Affect Condition, and Food Type.

Predictors		*B*	SE	*t*	p
Intercept		–0.46	0.07	–6.04	**<0.001**
Group		0.14	0.10	1.39	0.173
Affect Condition		–0.16	0.07	–2.16	**0.032**
Food Type		0.14	0.09	1.54	0.125
Group × Affect Condition		–0.23	0.10	–2.26	**0.026**
Group × Food Type		–0.29	0.12	–2.33	**0.022**
Affect Condition × Food Type		–0.12	0.09	–1.45	0.150
Group × Affect Condition × Food Type		0.28	0.12	2.36	**0.020**
*N*_subject_	46				
Observations	184				

Bold p-values indicate p < 0.05.

**Appendix 1—table 3.** Simple effects analyses comparing groups on attribute onset ($\tau _{s}$) difference scores.

Contrast	Estimate	SE	df	*t*	p
*Group difference in condition effect by food type*					
Condition effect for Low Fat foods	–0.23	0.10	132	–2.26	**0.026**
Condition effect for High Fat foods	0.05	0.10	132	0.51	0.614
*Group differences in food type effects by condition*					
Food type effects in Neutral condition	–0.29	0.13	132	–2.33	**0.022**
Food type effects in Negative condition	–0.01	0.03	132	–0.52	0.607
*Food type effects within groups and condition*					
Low fat vs. High fat (HC Neutral)	0.23	0.06	132	4.05	**<0.001**
Low fat vs. High fat (HC Negative)	0.29	0.05	132	5.64	**<0.001**
Low fat vs. High fat (BN Neutral)	0.22	0.06	132	3.61	**<0.001**
Low fat vs. High fat (BN Negative)	0.38	0.05	132	7.00	**<0.001**

Note. HC = healthy controls; BN = bulimia nervosa. Difference scores reflect food-type effects (high-fat minus low-fat) and condition effects (Negative minus Neutral). Bold p-values indicate p < 0.05.

**Appendix 1—table 4.** Linear mixed-effects regression analyzing tastiness attribute weights ($\omega _{taste}$) across Group, Affect Condition, and Food Type.

Predictors		*B*	SE	*t*	p
Intercept		0.57	0.04	13.55	**<0.001**
Group		–0.39	0.06	–6.86	**<0.001**
Affect Condition		–0.16	0.05	–2.96	**0.004**
Food Type		–0.23	0.06	–4.05	**<0.001**
Group × Affect Condition		0.18	0.07	2.46	**0.015**
Group × Food Type		–0.06	0.08	–0.83	0.410
Affect Condition × Food Type		0.01	0.05	0.21	0.834
Group × Affect Condition × Food Type		–0.10	0.07	–1.47	0.144
*N*_subject_	46				
Observations	184				

Bold p-values indicate p < 0.05.

**Appendix 1—table 5.** Simple effects analyses comparing groups on tastiness attribute weight ($\omega _{taste}$) difference scores.

Contrast	Estimate	SE	df	*t*	p
*Group difference by condition interaction*					
Neutral vs. Negative (HC – BN)	0.13	0.06	132	1.95	0.053
*Group differences within conditions*					
HC vs. BN (Neutral)	0.42	0.04	44	9.68	**<0.001**
HC vs. BN (Negative)	0.30	0.06	44	5.17	**<0.001**
*Condition effects within groups*					
Neutral vs. Negative (HC)	0.15	0.05	132	3.20	**0.002**
Neutral vs. Negative (BN)	0.03	0.04	132	0.61	0.542
*Food type effects within groups*					
Low-fat versus High-fat (HC)	0.22	0.05	132	4.29	**<0.001**
Low-fat versus High-fat (BN)	0.34	0.05	132	7.12	**<0.001**

Note. HC = healthy controls; BN = bulimia nervosa. Bold p-values indicate p < 0.05.

**Appendix 1—table 6.** Linear mixed-effects regression analyzing healthiness attribute weights ($\omega _{health}$) across Group, Affect Condition, and Food Type.

Predictors		*B*	SE	*t*	p
Intercept		–0.51	0.04	–12.14	**<0.001**
Group		0.38	0.06	6.73	**<0.001**
Affect Condition		0.09	0.05	1.82	0.071
Food Type		–0.04	0.07	–0.62	0.533
Group × Affect Condition		–0.13	0.07	–1.89	0.062
Group × Food Type		0.28	0.09	3.09	**0.002**
Affect Condition × Food Type		0.07	0.08	0.87	0.384
Group × Affect Condition × Food Type		–0.04	0.11	–0.34	0.735
*N*_subject_	46				
Observations	184				

Bold p-values indicate p < 0.05.

**Appendix 1—table 7.** Simple effects analyses comparing groups on healthiness attribute weight ($\omega _{health}$) difference scores.

Contrast	Estimate	SE	df	*t*	p
*Group Differences within conditions*					
HC vs. BN (Neutral)	–0.53	0.04	44	–12.02	**<0.001**
HC vs. BN (Negative)	–0.38	0.06	44	–6.42	**<0.001**
*Condition effects within groups*					
Neutral vs. Negative (HC)	–0.13	0.05	132	–2.50	**0.014**
Neutral vs. Negative (BN)	0.02	0.05	132	0.46	0.645
*Food type effects within groups*					
Low-fat versus High-fat (HC)	0.01	0.06	132	0.13	0.897
Low-fat versus High-fat (BN)	–0.26	0.06	132	–4.40	**<0.001**

Note. HC = healthy controls; BN = bulimia nervosa. Bold p-values indicate p < 0.05.

**Appendix 1—table 8.** Zero-inflated negative binomial model of symptom severity.

Outcome	Predictors			*B*	SE	*Z*	p
Subjective binge episodes	**Count model**	Intercept		–29.45	12.30	–2.40	**0.017**
		$\tau _{s\,\mathrm{Neu}\, {\rm LF}}$		–0.21	0.60	–0.36	0.722
		$\tau _{s\,\mathrm{Neu}\, {\rm HF}}$		1.48	1.29	1.14	0.254
		$\tau _{s\,\mathrm{Neg}\, {\rm LF}}$		1.09	4.02	–0.27	0.787
		$\tau _{s\,\mathrm{Neg}\, {\rm HF}}$		–46.39	16.56	–2.80	**0.005**
	**Zero-inflated model**	Intercept		–1.28	0.60	–2.12	**0.034**
		*N*_subject_	25				
		Observations	25				
Objective binge episodes	**Count model**	Intercept		5.69	4.20	1.36	0.175
		$\tau _{s\,\mathrm{Neu}\, {\rm LF}}$		0.47	0.28	1.69	0.091
		$\tau _{s\,\mathrm{Neu}\, {\rm HF}}$		0.16	0.59	0.27	0.790
		$\tau _{s\,\mathrm{Neg}\, {\rm LF}}$		0.99	3.03	0.33	0.745
		$\tau _{s\,\mathrm{Neg}\, {\rm HF}}$		0.07	4.62	0.02	0.998
	**Zero-inflated model**	Intercept		–21.27	8306.43	0.00	0.998
		*N*_subject_	25				
		Observations	25				

Note: Neu = Neutral affect condition; Neg = Negative affect condition; LF = Low-fat food; HF = High-fat food. Bold p-values indicate p < 0.05.

**Appendix 1—table 9.** Logistic mixed-effects regression analyzing food choice across Affect Condition and Food Type split by Group.

Predictors		*B*	SE	*Z*	p
Intercept		–0.29	0.18	–1.59	0.113
Group		–0.48	0.18	–2.63	**0.009**
Affect Condition		–0.02	0.09	–0.26	0.793
Food Type		–0.62	0.13	–4.58	**<0.001**
Group × Affect Condition		0.07	0.09	0.82	0.410
Group × Food Type		–0.27	0.13	–1.99	**0.047**
Affect Condition × Food Type		–0.03	0.05	–0.62	0.535
Group × Affect Condition × Food Type		–0.10	0.05	–1.91	0.056
*N*_subject_	46				
Observations	3271				

Bold p-values indicate p < 0.05.

**Appendix 1—table 10.** Logistic mixed-effects regression analyzing choice across Group and Affect Condition using tastiness and healthiness attribute ratings.

Predictors		*B*	SE	*Z*	p
Intercept		–0.60	0.24	–2.46	**0.014**
Group		–0.80	0.24	–3.36	**<0.001**
Affect Condition		0.05	0.20	0.26	0.797
Taste Rating [z]		2.93	0.26	11.40	**<0.001**
Health Rating [z]		1.49	0.24	6.29	**<0.001**
Group × Affect Condition		0.24	0.19	1.21	0.225
Taste × Group		–0.65	0.23	–2.78	**0.005**
Health × Group		0.64	0.23	2.77	**0.006**
Taste × Affect Condition		–0.45	0.17	–2.57	**0.010**
Health × Affect Condition		–0.10	0.17	–0.61	0.542
Taste × Group × Affect Condition		–0.03	0.13	–0.20	0.841
Health × Group × Affect Condition		0.04	0.16	0.24	0.812
*N*_subject_	46				
Observations	3271				

Bold p-values indicate p < 0.05.

**Appendix 1—table 11.** Logistic mixed-effects regression analyzing self-control across Group and Affect Condition.

Predictors		*B*	SE	*Z*	p
Intercept		–0.30	0.18	–1.68	0.093
Group		0.38	0.18	2.14	**0.033**
Affect Condition		0.08	0.12	0.70	0.486
Group × Affect Condition		0.15	0.12	1.27	0.204
*N*_subject_	46				
Observations	1118				

Bold p-values indicate p < 0.05.

**Appendix 1—table 12.** Linear mixed-effects regression analyzing log-transformed response times across Group and Affect Condition using tastiness and healthiness attribute ratings.

Predictors		*B*	SE	*Z*	p
Intercept		0.53	0.04	15.10	**<0.001**
Group		–0.02	0.04	–0.70	0.487
Affect Condition		–0.02	0.02	–1.16	0.250
Food Type		–0.03	0.01	–2.07	**0.044**
Choice		0.01	0.01	0.64	0.525
Group × Affect Condition		–0.02	0.02	–0.84	0.406
Group × Food Type		–0.02	0.01	–1.17	0.248
Affect Condition × Food Type		0.01	0.01	0.64	0.527
Group × Choice		0.03	0.01	2.44	**0.015**
Affect Condition × Choice		0.00	0.01	–0.50	0.959
Food Type × Choice		0.03	0.01	2.70	**0.007**
Group x Affect Condition × Food Type		–0.01	0.01	–0.64	0.524
Group x Affect Condition × Choice		–0.01	0.01	–1.26	0.208
Group x Food Type × Choice		–0.01	0.01	–0.65	0.513
Affect Condition × Food Type × Choice		0.01	0.01	0.75	0.455
Group x Affect Condition × Food Type × Choice		–0.01	0.01	–0.60	0.548
N _subject_	46				
Observations	3271				

Bold p-values indicate p < 0.05.

**Appendix 1—table 13.** Model fit metrics for alternative model specifications. All models were estimated with the time-varying drift rate but differed in which parameters varied by Food Type.

Model	Parameters per subject	Group	WAIC	Parameters varying with Food Type
M0	7	HC	5104	NaN
		BN	6510	
M1	9	HC	4886	$\omega _{health},\omega _{taste}$
		BN	6266	
M2	8	HC	4926	$\tau _{s}$
		BN	6298	
M3	10	HC	4874	$\tau _{s},\omega _{health},\omega _{taste}$
		BN	6135	

Note. $\omega _{health}=\text{health coefficient},\omega _{taste}=\text{taste coefficient},\tau _{s}=\text{relative starting time}$.

**Appendix 1—table 14.** Confusion matrix indicating frequency with which each candidate model was selected for each simulated model.

		Predicted			
Simulated	Model	M0	M1	M2	M3
	M0	1.00	0.00	0.00	0.00
	M1	0.00	1.00	0.00	0.00
	M2	0.00	0.00	0.00	1.00
	M3*	0.00	0.00	0.00	1.00

* symbol indicates winning model from model comparison based on empirical data.

**Appendix 1—table 15.** Analysis of variance using mixed effects models to assess deviance of M0 predicted choice frequencies compared to empirical data.

Terms		*F*	Df	p
Data source		18.36	1, 308	**<0.001**
Data source x Group		0.14	1, 308	0.709
Data source x Affect Condition		0.55	1, 308	0.458
Data source x Food Type		16.76	1, 308	**<0.001**
Data source x Group x Affect Condition		0.06	1, 308	0.808
Data source x Group x Food Type		1.32	1, 308	0.252
Data source x Affect Condition x Food Type		0.00	1, 308	0.964
Data source x Group x Affect Condition x Food Type		0.01	1, 308	0.919
*N*_subject_	46			
Observations	368			

Bold p-values indicate p < 0.05.

**Appendix 1—table 16.** Analysis of variance using mixed effects models to assess deviance of M1 predicted choice frequencies compared to empirical data.

Terms		*F*	Df	p
Data source		95.27	1, 308	**<0.001**
Data source × Group		4.73	1, 308	**0.030**
Data source × Affect Condition		0.10	1, 308	0.754
Data source × Food Type		67.78	1, 308	**<0.001**
Data source × Group × Affect Condition		0.89	1, 308	0.346
Data source × Group × Food Type		0.34	1, 308	0.563
Data source × Affect Condition × Food Type		0.18	1, 308	0.670
Data source × Group × Affect Condition × Food Type		0.60	1, 308	0.441
*N*_subject_	46			
Observations	368			

Bold p-values indicate p < 0.05.

**Appendix 1—table 17.** Analysis of variance using mixed effects models to assess deviance of M2 predicted choice frequencies compared to empirical data.

Terms		*F*	Df	p
Data source		14.08	1, 308	**<0.001**
Data source × Group		0.05	1, 308	0.827
Data source × Affect Condition		0.06	1, 308	0.803
Data source × Food Type		6.61	1, 308	**0.011**
Data source × Group × Affect Condition		0.20	1, 308	0.654
Data source × Group × Food Type		1.49	1, 308	0.223
Data source × Affect Condition × Food Type		0.27	1, 308	0.601
Data source × Group × Affect Condition × Food Type		0.05	1, 308	0.821
*N*_subject_	46			
Observations	368			

Bold p-values indicate p < 0.05.

**Appendix 1—table 18.** Analysis of variance using mixed effects models to assess deviance of M3 predicted choice frequencies compared to empirical data.

Terms		*F*	Df	p
Data source		8.84	1, 308	**0.003**
Data source × Group		0.00	1, 308	0.944
Data source × Affect Condition		0.92	1, 308	0.339
Data source × Food Type		1.70	1, 308	0.193
Data source × Group × Affect Condition		0.13	1, 308	0.720
Data source × Group × Food Type		0.70	1, 308	0.404
Data source × Affect Condition × Food Type		0.11	1, 308	0.741
Data source × Group × Affect Condition × Food Type		0.97	1, 308	0.326
*N*_subject_	46			
Observations	368			

Bold p-values indicate p < 0.05.

**Appendix 1—table 19.** Logistic mixed-effects regression assessing choice predictions from Model M0.

Predictors		*B*	SE	*Z*	p
Intercept		0.29	0.12	2.30	0.021
Group		–0.34	0.12	–2.74	**0.006**
Affect Condition		–0.08	0.07	–1.15	0.251
Food Type		–0.01	0.07	–0.19	0.848
Group × Affect Condition		0.03	0.07	0.39	0.695
Group × Food Type		–0.32	0.07	–4.34	**<0.001**
Affect Condition × Food Type		–0.05	0.05	–1.01	0.312
Group × Affect Condition × Food Type		–0.06	0.05	–1.27	0.205
*N*_subject_	46				
Observations	6542				

Bold p-values indicate p < 0.05.

**Appendix 1—table 20.** Logistic mixed-effects regression assessing choice predictions from Model M1.

Predictors		*B*	SE	*Z*	p
Intercept		0.89	0.15	6.12	**<0.001**
Group		–0.28	0.15	–1.93	0.053
Affect Condition		–0.08	0.07	–1.03	0.303
Food Type		0.42	0.07	6.35	**<0.001**
Group × Affect Condition		0.17	0.07	2.33	**0.020**
Group × Food Type		–0.22	0.07	–3.27	**0.001**
Affect Condition × Food Type		–0.04	0.04	–0.98	0.325
Group × Affect Condition × Food Type		0.02	0.04	0.60	0.552
*N*_subject_	46				
Observations	6542				

Bold p-values indicate p < 0.05.

**Appendix 1—table 21.** Logistic mixed-effects regression assessing choice predictions from Model M2.

Predictors		*B*	SE	*Z*	p
Intercept		0.28	0.14	2.05	**0.041**
Group		–0.42	0.14	–3.08	**0.002**
Affect Condition		–0.03	0.08	–0.39	0.695
Food Type		–0.16	0.11	–1.52	0.128
Group × Affect Condition		0.00	0.08	0.01	0.993
Group × Food Type		–0.36	0.11	–3.36	**<0.001**
Affect Condition × Food Type		–0.08	0.06	–1.37	0.170
Group x Affect Condition × Food Type		–0.07	0.06	–1.30	0.195
*N*_subject_	46				
Observations	6542				

Bold p-values indicate p < 0.05.

**Appendix 1—table 22.** Logistic mixed-effects regression assessing choice predictions from Model M3.

Predictors		*B*	SE	*Z*	p
Intercept		0.13	0.13	0.94	0.347
Group		–0.36	0.13	–2.68	**0.007**
Affect Condition		–0.11	0.07	–1.50	0.133
Food Type		–0.36	0.09	–4.16	**<0.001**
Group × Affect Condition		0.02	0.07	0.28	0.777
Group × Food Type		–0.27	0.09	–3.11	**0.002**
Affect Condition × Food Type		–0.09	0.04	–1.96	0.050
Group × Affect Condition × Food Type		0.03	0.04	0.58	0.562
*N*_subject_	46				
Observations	6542				

Bold p-values indicate p < 0.05.

**Appendix 1—table 23.** Linear mixed-effects regression assessing non-decision time ($\tau _{ND}$) across Group and Affect Condition.

Predictors		*B*	SE	*t*	p
Intercept		0.51	0.03	16.06	**<0.001**
Group		0.02	0.04	0.39	0.701
Affect Condition		0.02	0.03	0.74	0.464
Group × Affect Condition		–0.09	0.04	–2.19	**0.034**
*N*_subject_	46				
Observations	92				

Bold p-values indicate p < 0.05.

**Appendix 1—table 24.** Linear mixed-effects regression assessing boundary separation (α) across Group and Affect Condition.

Predictors		*B*	*SE*	*t*	p
Intercept		3.46	0.10	33.55	**<0.001**
Group		–0.40	0.14	–2.84	**0.007**
Affect Condition		–0.37	0.09	–4.26	**<0.001**
Group × Affect Condition		0.23	0.12	1.94	0.058
*N*_subject_	46				
Observations	92				

Bold p-values indicate p < 0.05.

**Appendix 1—table 25.** Linear mixed-effects regression assessing starting point (*z*) across Group and Affect Condition.

Predictors		*B*	SE	*t*	p
Intercept		0.41	0.02	20.13	**<0.001**
Group		–0.01	0.03	–0.48	0.634
Affect Condition		–0.02	0.02	–1.01	0.317
Group × Affect Condition		0.03	0.03	0.98	0.331
*N*_subject_	46				
Observations	92				

Bold p-values indicate p < 0.05.

**Appendix 1—table 26.** Linear mixed-effects regression assessing the Anger POMS subscale as a function of Group, Affect Condition, and Timing.

Predictors		*B*	SE	*t*	p
Intercept		0.57	1.77	0.32	0.748
Group		7.59	2.40	3.16	**0.002**
Affect Condition		0.00	1.33	0.00	1.000
Timing		0.00	1.33	0.00	1.000
Group × Affect Condition		–2.28	1.80	–1.26	0.208
Group × Timing		–1.01	1.83	–0.55	0.581
Affect Condition × Timing		5.71	1.88	3.04	**0.003**
Group × Affect Condition × Timing		0.66	2.57	0.26	0.798
*N*_subject_	46				
Observations	184				

Bold p-values indicate p < 0.05.

**Appendix 1—table 27.** Linear mixed-effects regression assessing the Confusion POMS subscale as a function of Group, Affect Condition, and Timing.

Predictors		*B*	SE	*t*	p
Intercept		2.99	0.94	3.17	**0.002**
Group		6.07	1.28	4.75	**<0.001**
Affect Condition		–0.74	0.68	–1.08	0.282
Timing		–0.84	0.68	–1.22	0.224
Group × Affect Condition		–0.44	0.92	–0.48	0.630
Group × Timing		1.14	0.93	1.23	0.221
Affect Condition × Timing		2.30	0.95	2.42	**0.017**
Group x Affect Condition × Timing		–0.92	1.30	–0.71	0.482
*N*_subject_	46				
Observations	173				

Bold p-values indicate p < 0.05.

**Appendix 1—table 28.** Linear mixed-effects regression assessing the Depression POMS subscale as a function of Group, Affect Condition, and Timing.

Predictors		*B*	SE	*t*	p
Intercept		0.82	2.57	0.32	0.750
Group		16.06	3.47	4.63	**<0.001**
Affect Condition		–0.34	1.66	–0.21	0.836
Timing		–0.49	1.63	–0.30	0.765
Group × Affect Condition		–4.01	2.23	–1.80	0.074
Group × Timing		–0.20	2.23	–0.09	0.928
Affect Condition × Timing		5.58	2.29	2.44	**0.016**
Group × Affect Condition × Timing		3.56	3.13	1.14	0.258
*N*_subject_	46				
Observations	174				

Bold p-values indicate p < 0.05.

**Appendix 1—table 29.** Linear mixed-effects regression assessing the Fatigue POMS subscale as a function of Group, Affect Condition, and Timing.

Predictors		*B*	SE	*t*	p
Intercept		3.52	1.33	2.65	**0.010**
Group		6.04	1.81	3.34	**0.001**
Affect Condition		–1.26	0.95	–1.34	0.183
Timing		0.48	0.93	0.51	0.610
Group × Affect Condition		1.82	1.27	1.43	0.154
Group × Timing		–0.98	1.28	–0.77	0.443
Affect Condition × Timing		0.74	1.33	0.56	0.578
Group × Affect Condition × Timing		–0.35	1.80	–0.20	0.845
*N*_subject_	46				
Observations	181				

Bold p-values indicate p < 0.05.

**Appendix 1—table 30.** Linear mixed-effects regression assessing the Tension POMS subscale as a function of Group, Affect Condition, and Timing.

Predictors		*B*	SE	*T*	p
Intercept		2.76	1.47	1.88	0.066
Group		8.76	2.00	4.38	**<0.001**
Affect Condition		0.05	0.79	0.06	0.952
Timing		0.10	0.79	0.12	0.904
Group × Affect Condition		–1.57	1.07	–1.47	0.145
Group × Timing		–2.50	1.07	–2.34	**0.021**
Affect Condition × Timing		1.86	1.11	1.67	0.098
Group × Affect Condition × Timing		3.82	1.51	2.53	**0.013**
*N*_subject_	46				
Observations	184				

Bold p-values indicate p < 0.05.

**Appendix 1—table 31.** Linear mixed-effects regression assessing the Vigor POMS subscale as a function of Group, Affect Condition, and Timing.

Predictors		*B*	SE	*t*	p
Intercept		17.52	1.33	13.14	**<0.001**
Group		–10.40	1.82	–5.73	**<0.001**
Affect Condition		0.88	0.98	0.90	0.372
Timing		–0.57	0.96	–0.59	0.554
Group × Affect Condition		0.87	1.34	0.65	0.518
Group × Timing		0.48	1.34	0.36	0.720
Affect Condition × Timing		–4.93	1.37	–3.58	**<0.001**
Group × Affect Condition × Timing		1.30	1.90	0.68	0.495
*N*_subject_	46				
Observations	176				

Bold p-values indicate p < 0.05.

**Appendix 1—table 32.** Linear regression models of negative urgency.

Parameter	Predictors		*B*	SE	*t*	p
Attribute onset ($\tau _{s}$)	Intercept		–2.18	3.61	–0.61	0.552
	Neutral_LF_		0.17	0.22	0.76	0.456
	Neutral_HF_		0.13	0.47	0.28	0.779
	Negative _LF_		1.12	2.52	0.45	0.661
	Negative _HF_		–8.37	4.15	–2.02	0.057
	*N*_subject_	25				
	Observations	25				
Tastiness weight ($\omega _{Taste}$)	Intercept		2.92	0.15	19.61	**<0.001**
	Neutral _LF_		0.26	0.46	0.57	0.578
	Neutral _HF_		0.73	0.57	1.28	0.215
	Negative _LF_		–0.51	0.47	–1.08	0.291
	Negative _HF_		–0.17	0.47	–0.36	0.724
	*N*_subject_	25				
	Observations	25				
Healthiness weight ($\omega _{Health}$)	Intercept		2.86	0.11	26.01	**<0.001**
	Neutral _LF_		–0.59	0.36	–1.62	0.122
	Neutral _HF_		–0.42	0.31	–1.38	0.184
	Negative _LF_		0.65	0.39	1.66	0.113
	Negative _HF_		0.27	0.27	1.00	0.330
	*N*_subject_	25				
	Observations	25				

Bold p-values indicate p < 0.05.

**Appendix 1—table 33.** Linear regression models of the restraint subscale of EDE-Q.

Parameter	Predictors		*B*	SE	*t*	p
Attribute onset ($\tau _{s}$)	Intercept		13.81	14.00	0.99	0.336
	Neutral _LF_		0.58	0.87	0.66	0.515
	Neutral _HF_		1.50	1.83	0.82	0.424
	Negative _LF_		1.45	9.77	0.15	0.883
	Negative _HF_		11.87	16.09	0.74	0.469
	*N*_subject_	25				
	Observations	25				
Tastiness weight ($\omega _{Taste}$)	Intercept		4.45	0.51	8.80	**<0.001**
	Neutral _LF_		–2.67	1.55	–1.72	0.101
	Neutral _HF_		1.46	1.93	0.76	0.457
	Negative _LF_		–1.28	1.61	–0.79	0.437
	Negative _HF_		–0.28	1.58	–0.17	0.863
	*N*_subject_	25				
	Observations	25				
Healthiness weight ($\omega _{Health}$)	Intercept		3.99	0.42	9.42	**<0.001**
	Neutral _LF_		1.82	1.40	1.30	0.209
	Neutral _HF_		0.08	1.19	0.07	0.949
	Negative _LF_		1.40	1.51	0.93	0.366
	Negative _HF_		0.45	1.03	0.44	0.668
	*N*_subject_	25				
	Observations	25				

Bold p-values indicate p < 0.05.
