# Supplementary material for: Negative affect influences the computations underlying food choice in bulimia nervosa
Source: eLife. 2026 Oct 1;14:RP105146. doi: 10.7554/eLife.105146 (PMC13630384; doi:10.7554/eLife.105146)
Supplement: Peer review history

# Editor report: eLife Assessment

**DOI:** 10.7554/eLife.105146.5.sa0

**Authors:** Andreea Oliviana Diaconescu

**Affiliations:** University of Toronto Canada

**Keywords:** Solid, Valuable

This study makes a **valuable** contribution to understanding how negative affect shapes food-choice decision making in bulimia nervosa by using a mechanistic drift diffusion model to quantify the weighting and temporal integration of tastiness and healthiness attributes. The approach is **solid** and has clear potential to advance understanding of the decision processes underlying pathological food choices. The evidence is strengthened by the randomised crossover design and appropriate statistical analyses. The results are consistent across different analytic approaches, increasing confidence in the robustness of the findings.

# Referee report: Reviewer #1 (Public review)

**DOI:** 10.7554/eLife.105146.5.sa1

Summary:

Using a computational modeling approach based on the Drift and Diffusion Model (DDM) introduced by Ratcliff and McKoon in 2008, the article by Shevlin and colleagues investigates whether there are differences between neutral and negative emotional states in:

(1) The timings of the integration in food choices of the perceived healthiness and tastiness of food options in individuals with bulimia nervosa and healthy participants

(2) The weighting of the perceived healthiness and tastiness of these options.

Strengths:

By looking at the mechanistic part of the decision process, the approach has potential to improve the understanding of pathological food choices.

Comments on revised version:

I went carefully through the answers of the authors to my last concerns - they answered all my points. I am grateful that they obtained consistent results with the different analyses.

# Author comment: Author response

**DOI:** 10.7554/eLife.105146.5.sa2

**Authors:** Blair RK Shevlin, Loren Gianini, Joanna Steinglass, Karin Foerde, E Caitlin Lloyd, Kelsey Hagan, Laura A Berner

**Affiliations:** Icahn School of Medicine at Mount Sinai New York United States; Columbia University Medical Center New York United States; Columbia University Medical Center New York United States; New York State Psychiatric Institute New York United States; Columbia University Irving Medical Center New York United States; Virginia Commonwealth University Medical Center Richmond United States; Icahn School of Medicine at Mount Sinai New York United States

The following is the authors’ response to the previous reviews

**eLife Assessment**

This study makes a valuable contribution to understanding how negative affect shapes food-choice decision making in bulimia nervosa by leveraging a mechanistic drift diffusion model to quantify the weighting of tastiness and healthiness attributes. The evidence is solid, supported by a randomized crossover design and generally appropriate statistical analyses. However, the interpretability of the findings is limited by ambiguities in the affect manipulation, particularly regarding whether neutral and negative inductions yielded reliably distinct affective states at the time of task performance in the bulimia nervosa group. Consequently, session-related differences in model parameters cannot be unequivocally attributed to negative affect rather than to uncontrolled state or contextual factors, and clearer separation of affective conditions alongside analyses aligned with the paired data structure would strengthen the conclusions.

We thank the Editor and Reviewers for their careful summary of the study's strengths and for their constructive feedback.

The eLife Assessment identified two specific limitations that qualified the strength of evidence:

(1) ambiguity regarding whether the two affect inductions yielded reliably distinct affective states in the BN group at the time of task performance, and (2) analyses that were not fully aligned with the paired data structure. We have directly addressed both concerns in this revision. We provide explicit statistical evidence confirming that neutral and negative inductions yielded distinct affective states in the bulimia nervosa group; and we have re-analyzed all DDM parameters using updated mixed-effects regressions with an unstructured covariance matrix that appropriately accounts for the paired data structure. For completeness, we have also added the requested difference-in-difference analysis. Both approaches yielded conclusions consistent with those originally reported.

In light of these revisions, we would be grateful if the Editorial Team would consider whether the strength of evidence rating might be updated from "solid" to "convincing." All changes in the revised manuscript are marked in blue.

**Public Reviews:**

**Reviewer #1 (Public review):**

Summary:

Using a computational modeling approach based on the Drift and Diffusion Model (DDM) introduced by Ratcliff and McKoon in 2008, the article by Shevlin and colleagues investigates whether there are differences between neutral and negative emotional states in:

(1) The timings of the integration in food choices of the perceived healthiness and tastiness of food options in individuals with bulimia nervosa (BN) and healthy participants (2) The weighting of the perceived healthiness and tastiness of these options.

Strengths:

By looking at the mechanistic part of the decision process, the approach has potential to improve the understanding of pathological food choices.

Weaknesses:

I thank the authors for revising their manuscript.

I still notice that the authors did not go through their manuscript to look for wordings refering to a prediction interpretation of their results while I already highlighted the inappropriateness of this wording in my two first rounds of reviews: e.g. there is still "we used zero-inflated negative binomial models to predict the three-month frequency" and I can find other statements like this. The design of their study does not allow such claims.

We thank the Reviewer for identifying cases where the term “predicted” may mislead readers about the causal nature of our claims. We have made the following edits (changes are italicized):

Methods (lines 516-518): “For these exploratory analyses, we used negative binomials to test the association between parameter estimates and the three-month frequency of retrospectively reported Objective Binge Episodes (OBE) and Subjective Binge Episodes (SBE).”

Figure 5 (lines 881-882): “Affect-induced changes in information onset were associated with more frequent subjective binge episodes.

The authors answered my major concern regarding the experimental induction towards a negative or a neutral state before running the food decision task. My concern is: BN patients already seemed to be already in a high negative state before undergoing the neutral induction, while these patients are in a lower negative state before undergoing the negative induction. It is therefore not surprising that patients seem to report a similar level of negative state after the two inductions (according to the figure of the authors' previous article). Of note is that the additional analysis the authors ran within the BN group only provides a significant result: this result shows that there has been an induction but does not rule out that patients were in the exact same magnitude of negative state to perform the task as the figure in their previously published article suggests it. The major issue is to show that:

(1) As compared to the neutral induction, there has been a higher variation in negative state after as compared to before the negative induction.

(2) The magnitude of the negative state after the negative induction is higher than the magnitude of the negative state after the neutral induction.

The first point shows that the induction worked. The second point shows that the participants are in two distinct states. Without showing the second point, it may be possible that one induction increases the negative state of participants to the same level as the one of the second induction that has not increased anything.

Within this context, how is it possible to associate, in patients, a difference in the DDM between the two sessions to a negative state (which is one of the main focus of the article) rather than to another parameter that has not been captured? A similar situation would be in an experiment studying the consequence of stress, a stressfull induction over relaxed participants attending the lab has high chances to raise the level of stress of those participants to the same level as the one that the same participants would experience after a neutral induction when these participants attend the lab with an already high level of stress. In that case, would it be approrpiate to claim that a difference at a task performed after the induction would be related to stress while the participants would be at the same level of stress when performing the task despite the fact that the induction worked ?

In the experiment performed by the authors, the additional analysis to perform would be a paired sample t-test (or the appropriate non-parametric test) to check whether the magnitude of negative state of BN patients was different between the negative and neutral conditions after the induction only. If not, associating the difference at the DDM with negative states in BN is highly misleading.

We thank the Reviewer for pressing on this point, and we apologize that our previous response did not make this sufficiently explicit. We agree with the Reviewer that two things must be demonstrated: (1) that the negative induction produced a greater change in negative affect than the neutral induction, and (2) that the magnitude of post-induction negative affect was higher following the negative induction than the neutral induction. We had included the results of analyses addressing both points in the Supplementary Materials of our previous submission, but we appreciate that we had not made this clear in our response.

Regarding point (1), the mixed-effects model in Supplementary Table S1 yielded a significant Affect Condition × Timing interaction (β = 20.43, SE = 6.35, t = 3.22, p = 0.002), confirming that negative affect increased significantly more from pre- to post-induction in the negative condition than in the neutral condition. This is further supported by within-BN-group analyses in the Supplementary Materials: the negative affect induction produced a large, significant increase in negative affect (mean difference = 20.36, SE = 4.21, t = 4.84, p < 0.0001, Cohen's d = 0.97), whereas the neutral induction was not associated with a significant change in negative affect (mean difference = 7.16, SE = 4.21, t = 1.70, p = 0.327, Cohen's d = 0.34).

Regarding point (2), we directly compared post-induction negative affect between conditions within the BN group, as requested by the Reviewer. The magnitude of negative affect was significantly higher following the negative mood induction than after the neutral mood induction (mean difference = 17.40, SE = 4.21, t = 4.13, p = 0.0003, Cohen's d = 0.83). This large effect size confirms that participants with BN were in meaningfully distinct affective states when performing the food decision task under the two conditions.

Together, these analyses establish (1) that the induction worked as intended, and (2) that the two post-induction states were both statistically and practically distinct. We have added explicit language to the manuscript to make both of these points clear (lines: 181-185):

Critically, post-induction negative affect within the BN group was significantly higher following the negative affect induction than after the neutral affect induction (mean difference = 17.40, SE = 4.21, t = 4.13, p < 0.001, Cohen's d = 0.83; see Supplementary Materials for full details), confirming that BN participants completed the food decision task under meaningfully distinct affective states across the two sessions.

I read carefully the authors' answer related to mixed models: they claim that mixed models take into account correlations within their repeated data. The specification of the structure of the covariance matrix allows to control only partly for that. I notice that the authors did not specify the structure of that matrix: the article they refer to justify the appropriateness of their analyses is not adapted. The specification of the structure of the covariance matrix needs to address, in a mixed model, the difference in handling 4 repeated data per participants that cannot be paired as compared to 4 repeated data that can be paired (two per session with one before and one after the neutral or negative priming sessions, if I count right). Of note is that a covariance structure that is left free of constraint for the fit of the model does not capture appropriately the pairing of the data: it has all chances to capture the covariance in a different way. And a covariance structure that has constraints has more chances to lead to a model that cannot be estimated because of an absence of convergence of the algorithms.

By the way, a single two-sample t-test (or a Mann-Whitney test if appropriate), and not a set of multiple paired-sample t-test as the authors suggest, would answer the goal of the authors to test for what they call the three-way interaction in their comment. This test would be performed between the two groups of participants (BN/controls) with the computation for each participant separately: (assessment after neutral induction-assessment before neutral induction)-(assessment after negative induction-assessment before negative induction). This analysis answers points 1, 2 and 4 they raise together with my point of controlling for the paired data. I would have agreed with their choice of a mixed model if they had an unbalanced dataset within each participant.

We thank the Reviewer for this clarification, and we apologize that our previous response did not adequately distinguish between two different sets of analyses: (1) analyses of DDM parameter estimates, which involved four observations per participant (2 affect conditions × 2 food types); (2) trial-level analyses of choice and response time behavior, where each participant contributed many trials per condition and the dataset is genuinely unbalanced across participants due to trial exclusions – precisely the situation where mixed-effects models with participant-level random slopes are appropriate. The concern about covariance structure applies specifically to the DDM parameter analyses, but does not apply to our trial-level analyses.

We also want to clarify a point about the task design that may have caused confusion. The Food Choice Task was administered only once per session, after the mood induction (i.e., once after negative mood induction, and once after neutral mood induction). As detailed in Figure 1, the task was not completed pre-induction. The four observations per participant in the DDM parameter analyses therefore reflect 2 affect conditions × 2 food types assessed within each condition, not a pre/post structure. This does not change how we address the concern about covariance structure, as there is still a nested feature of interest (food type within condition), but we wanted to correct this misunderstanding explicitly.

For the DDM parameter analyses, we agree with the Reviewer that the original random effects structure did not adequately account for the paired nature of the four within-person observations.

We have addressed this in two ways.

First, we re-estimated the mixed model specifying an unstructured covariance matrix using the nlme package, which places no constraints on the correlation pattern among the four withinperson observations. We acknowledge the Reviewer's point that an unconstrained covariance matrix is not guaranteed to recover the within-session pairing structure. We explored whether a more constrained specification would be preferable. Specifically, we tested a nested random effect of affect condition within subject, which would directly encode the pairing of Low-Fat and High-Fat observations within each session. However, this model failed to converge. This is not a numerical issue but a fundamental identification problem: with only two observations per session per subject, the session-level and residual variance components cannot be separately estimated. We therefore selected the unstructured model as a more conservative option. Importantly, even if the unstructured model does not explicitly encode the pairing, it is a more general mathematical formula which would not impose incorrect constraints on the correlation structure.

Consistent with our original findings, the mixed model with an unstructured covariance matrix yielded a significant three-way interaction (Group × Condition × Food Type: β = 0.28, SE = 0.12, t = 2.36, p = 0.020). All simple effects analyses have been updated to reflect the models with this covariance structure, and these are reported in the updated Supplementary Tables.

Second, following the Reviewer's suggestion (adapted to the actual design structure, in which the Food Choice Task was administered once per session after the mood induction rather than before and after), we computed a difference-in-difference score for each participant's relative attribute onset parameter (τ_s_) following the affect inductions: (negative condition, high-fat − negative condition, low-fat) − (neutral condition, high-fat − neutral condition, low-fat). This score directly encodes the paired structure by construction, bypassing the covariance specification problem entirely. Consistent with the Reviewer's recommendation to use a non-parametric test where appropriate, we used a Wilcoxon rank-sum test (equivalent to Mann-Whitney U) to compare these difference scores between groups. The results confirmed that BN participants showed significantly larger food-type-specific changes in τs following negative affect induction relative to HC (W = 156, p = 0.018). We then applied this approach to all other DDM parameters (i.e., ω_taste_, ω_health_, α, τ_ND_, and z), and report these results alongside updated mixed-effects model results in the Supplementary Materials. The conclusions drawn from the difference-in-difference analyses were consistent with those from the mixed-effects models across all parameters.

Both approaches converge on the same conclusion and we report both sets of complementary results in the manuscript: the updated mixed-effects models address the full factorial design in a single framework, while the added difference-in-difference analyses explicitly resolve the covariance specification problem by encoding the paired structure directly into each participant’s score, as the Reviewer recommended.

**Reviewer #2 (Public review):**

Summary:

Binge eating is often preceded by heightened negative affect, but the specific processes underlying this link are not well-understood. The purpose of this manuscript was to examine whether affect state (neutral or negative mood) impacts food choice decision-making processes that may increase likelihood of binge eating in individuals with bulimia nervosa (BN). The researchers used a randomized crossover design in women with BN (n=25) and controls (n=21), in which participants underwent a negative or neutral mood induction prior to completing a food-choice task. The researchers found that despite no differences in food choices in the negative and neutral conditions, women with BN demonstrated a stronger bias toward considering the 'tastiness' before the 'healthiness' of the food after the negative mood induction.

Strengths:

The topic is important and clinically relevant and methods are sound. The use of computational modeling to understand nuances in decision-making processes and how that might relate to eating disorder symptom severity is a strength of the study.

Weaknesses:

Sample size was relatively small, and participants were all women with BN, which limits generalizability of findings to the larger population of individuals who engage in binge eating. It is likely that the negative affect manipulation was weak and may not have been potent enough to change behavior. These limitations are adequately noted in the discussion.

We thank the reviewer for their thorough description of the strengths and weaknesses of this study.
